# The orphan histidine protein kinase SgmT is a c-di-GMP receptor and regulates composition of the extracellular matrix together with the orphan DNA binding response regulator DigR in *Myxococcus xanthus*

**DOI:** 10.1111/j.1365-2958.2012.08015.x

**Published:** 2012-03-06

**Authors:** Tobias Petters, Xin Zhang, Jutta Nesper, Anke Treuner-Lange, Nuria Gomez-Santos, Michael Hoppert, Urs Jenal, Lotte Søgaard-Andersen

**Affiliations:** 1 Max Planck Institute for Terrestrial MicrobiologyKarl-von-Frisch Str. 10, 35043 Marburg, Germany; 2 Biozentrum of the University of BaselKlingelbergstrasse 50, CH-4054 Basel, Switzerland; 3 Institute of Microbiology & Genetics, Georg-August-Universität Göttingen37077 Göttingen, Germany

## Abstract

In *Myxococcus xanthus* the extracellular matrix is essential for type IV pili-dependent motility and starvation-induced fruiting body formation. Proteins of two-component systems including the orphan DNA binding response regulator DigR are essential in regulating the composition of the extracellular matrix. We identify the orphan hybrid histidine kinase SgmT as the partner kinase of DigR. In addition to kinase and receiver domains, SgmT consists of an N-terminal GAF domain and a C-terminal GGDEF domain. The GAF domain is the primary sensor domain. The GGDEF domain binds the second messenger bis-(3′-5′)-cyclic-dimeric-GMP (c-di-GMP) and functions as a c-di-GMP receptor to spatially sequester SgmT. We identify the DigR binding site in the promoter of the *fibA* gene, which encodes an abundant extracellular matrix metalloprotease. Whole-genome expression profiling experiments in combination with the identified DigR binding site allowed the identification of the DigR regulon and suggests that SgmT/DigR regulates the expression of genes for secreted proteins and enzymes involved in secondary metabolite synthesis. We suggest that SgmT/DigR regulates extracellular matrix composition and that SgmT activity is regulated by two sensor domains with ligand binding to the GAF domain resulting in SgmT activation and c-di-GMP binding to the GGDEF domain resulting in spatial sequestration of SgmT.

## Introduction

A fundamental property of bacterial cells is their ability to respond to changes in the extracellular and intracellular environments. In this context, specific inputs are coupled to specific outputs by dynamically interacting signalling proteins organized in regulatory pathways. In order to ensure an optimal coupling between input and output, the specificity in the input–output coupling is essential. Despite the multitude of cues sensed by bacteria, the signal transduction modalities involved centre on a few systems including two-component signal transduction systems (TCS) (Galperin, 2005) in which information transfer depends on phosphorylation/dephosphorylation (Stock *et al*., 2000). Generally, the number of TCS proteins in bacterial species scales with genome size (Galperin, 2005, Galperin, 2006) and some species contain several hundreds of these proteins raising the question how specificity within a given organism is accomplished.

TCS are composed of a histidine protein kinase and a response regulator (Stock *et al*., 2000). Typically, the kinases have an N-terminal sensor domain, which is responsible for detecting a particular stimulus, and a conserved kinase domain. Response regulators are either single-domain proteins consisting of the conserved receiver domain or multi-domain proteins consisting of a receiver domain and a variable output domain. Upon stimulus detection, the kinase autophosphorylates on a conserved His residue. The phosphoryl group is subsequently transferred to a conserved Asp residue in the receiver domain of the cognate response regulator resulting in its activation. Cognate TCS partners display a kinetic preference for each other *in vitro* (Skerker *et al*., 2005; Yamamoto *et al*., 2005; Biondi *et al*., 2006), and it has been argued that the molecular mechanism that ensures specificity in TCS signalling and precludes deleterious cross-talk *in vivo* depends on the kinetic preference of cognate TCS protein pairs for each other (Skerker *et al*., 2005; Laub and Goulian, 2007).

Typically, cognate TCS partners are encoded by adjacent genes and easily recognized (Stock *et al*., 2000). However, TCS genes are also found as orphan genes. For instance in *Caulobacter crescentus* 57% of the 106 TCS genes are orphans (Skerker *et al*., 2005), in *Bacillus subtilis* 14% of the 36 HPKs are orphans (Fabret *et al*., 1999), and in *Myxococcus xanthus* 55% of the more than 250 TCS genes are orphans (Shi *et al*., 2008; Whitworth and Cock, 2008). This genetic organization constitutes a formidable challenge in order to establish the connectivity of TCS proteins in cognate partners.

*Myxococcus xanthus* is a Gram-negative bacterium that organizes into two distinct patterns depending on the nutritional status of cells (Konovalova *et al*., 2010). In the presence of nutrients, cells organize into spreading colonies and in the absence of nutrients cells aggregate to form fruiting bodies inside which the rod-shaped cells differentiate to spores. Formation of spreading colonies as well as fruiting bodies depends on motility. *M. xanthus* cells move by gliding motility using two motility systems that display different selective advantages on different surfaces (Shi and Zusman, 1993). The A-motility system provides cells with the ability to move as single cells. In this system, mechanical force is generated in focal adhesion complexes that are distributed along the length of the cells (Mignot *et al*., 2007; Nan *et al*., 2011; Sun *et al*., 2011). The S-motility system depends on type IV pili (T4P) and is the equivalent of twitching motility in *Neisseria* and *Pseudomonas* species (Leonardy *et al*., 2008). T4P are thin filaments, several microns in length (Craig *et al*., 2004) and undergo cycles of extension and retractions (Merz *et al*., 2000; Sun *et al*., 2000; Skerker and Berg, 2001). While extension does not generate a force sufficient to move a cell, a force exceeding 100 pN per T4P is generated during retractions (Maier *et al*., 2002; Clausen *et al*., 2009). In *M. xanthus*, T4P-dependent motility depends on direct cell–cell contacts and this contact-dependence is thought to rely on exopolysaccharides (EPS) in the extracellular matrix (ECM) triggering retraction of T4P (Li *et al*., 2003).

*Myxococcus xanthus* cells are covered by an ECM composed of EPS and proteins in a 1:1 ratio (Behmlander and Dworkin, 1994a). Accumulation of ECM is a highly regulated process involving several TCS proteins and lack of these proteins causes defects in cell–cell cohesion, signal transduction, T4P-dependent motility and fruiting body formation (Konovalova *et al*., 2010). The dominant ECM protein is the metalloprotease FibA, which is important for aggregation of cells into fruiting bodies and regulation of motility in response to dilauroyl phosphatidylethanolamine (Kearns *et al*., 2002). Moreover, proteins of unknown function have been identified in the ECM (Curtis *et al*., 2007). The orphan DNA binding response regulator DigR was originally identified because a Δ*digR* mutation causes a defect in T4P-dependent motility and fruiting body formation (Overgaard *et al*., 2006). Based on the observation that a *digR* mutant assembles T4P, accumulates increased amounts of EPS, and decreased amounts of FibA it was suggested that the primary defect in a *digR* mutant is the abnormal composition of ECM and that the motility defect is indirect and caused by the increased EPS accumulation (Overgaard *et al*., 2006). Genetic evidence suggest that DigR depends on phosphorylation for full activity (Overgaard *et al*., 2006); however, the cognate kinase has not been identified. Here, we used a candidate approach to identify the cognate kinase of the orphan, DNA binding response regulator DigR in *M. xanthus* (Overgaard *et al*., 2006).

Recently, bis-(3′-5′)-cyclic-dimeric-GMP (c-di-GMP) has emerged as an important second messenger in bacteria (Jenal and Malone, 2006; Hengge, 2009). In general, c-di-GMP controls the switch from a planktonic, motile lifestyle to a surface-associated, sessile lifestyle by regulating EPS accumulation, surface adhesion, motility, subcellular localization of proteins and cell-surface protein localization (Jenal and Malone, 2006; Hengge, 2009). c-di-GMP is synthesized by diguanylate cyclases (DGCs) and degraded by phosphodiesterases (PDEs). DGCs share in common the so-called GGDEF domain named after a conserved amino acid motif in the active (A) site (Schirmer and Jenal, 2009). GGDEF domains often also contain a second c-di-GMP binding site, the so-called I-site (Schirmer and Jenal, 2009). GGDEF domains are subdivided based on the presence and absence of the A- and I-sites (Schirmer and Jenal, 2009): domains with intact A- and I-sites have DGC activity and are subject to non-competitive product inhibition by c-di-GMP binding to the I-site whereas domains with a degenerate A-site and an intact I-site may function as c-di-GMP receptors (Duerig *et al*., 2009). Similarly, PDEs share in common either an EAL or an HD-GYP domain, both named after a conserved amino acid motif in their active sites (Schirmer and Jenal, 2009). Proteins involved in c-di-GMP metabolism and regulation are ubiquitous with some species containing more than 50 proteins with GGDEF and EAL domains (Galperin, 2005; Hengge, 2009). The identification of several c-di-GMP receptors is beginning to illuminate how signalling by c-di-GMP can elicit specific responses. c-di-GMP receptors include PopA in *C. crescentus* with a GGDEF domain with a degenerate A-site and an intact I-site (Duerig *et al*., 2009), LapD in *Pseudomonas fluorescens* with an EAL domain without the signature EAL motif (Newell *et al*., 2009), PelD in *P. aeruginosa* that is unrelated to GGDEF domains but contains an I-site like sequence (Lee *et al*., 2007), transcriptional regulators (Hickman and Harwood, 2008; Chin *et al*., 2010; Krasteva *et al*., 2010; Wilksch *et al*., 2011), a polynucleotide phosphorylase RNA processing enzyme in *Escherichia coli* (Tuckerman *et al*., 2011), riboswitches (Sudarsan *et al*., 2008) and PilZ domains involved in regulating flagellar motility in *E. coli* and *Salmonella typhimurium* (Ryjenkov *et al*., 2006; Boehm *et al*., 2010; Paul *et al*., 2010).

Here, we aimed to identify the cognate histidine kinase of DigR to understand its function in ECM regulation. Using a candidate approach, we identify the orphan histidine kinase SgmT as the partner kinase of DigR. Our data suggest that an N-terminal GAF domain in SgmT is the primary sensor domain and that a C-terminal GGDEF domain is a receptor for c-di-GMP and functions to spatially sequester SgmT upon c-di-GMP binding. Moreover, we show that DigR directly regulates expression of the *fibA* gene and likely also that of genes encoding secreted proteins of unknown functions and encoding enzymes involved in secondary metabolite synthesis.

## Results

### *sgmT* and *digR* act in the same pathway

**Fig. 1:**
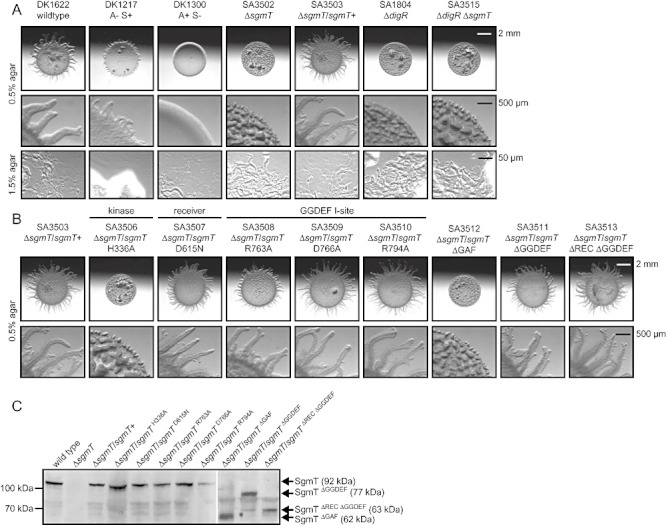
SgmT and DigR are required for T4P-dependent motility. A and B. Motility phenotype of Δ*sgmT* and Δ*digR* mutants. Strains were incubated at 32°C for 24 h on 0.5% or 1.5% agar supplemented with 0.5% CTT, and visualized with a Leica MZ86 stereomicroscope. C. Immunoblot analysis of the accumulation of SgmT variants. Total cell lysates from exponentially growing cells were separated by SDS-PAGE, and probed with α-SgmT antibodies. Proteins from the same numbers of cells (5 × 10^7^) were loaded per lane. Positions of SgmT variants and molecular size markers are indicated.

To identify the cognate DigR partner kinase, we hypothesized that the corresponding gene would be orphan and that its inactivation would also cause a defect in T4P-dependent motility. The *sgmT* gene (MXAN4640) fulfils these two criteria, it is orphan (Shi *et al*., 2008) and previously suggested to be important for T4P-dependent motility (Youderian and Hartzell, 2006). To test genetically whether *sgmT* and *digR* act in the same pathway, an in-frame deletion of *sgmT* (Δ*sgmT*) was generated. In *M. xanthus*, A-motility is favoured on 1.5% agar and T4P-dependent motility on 0.5% agar. Using these two conditions, the Δ*digR* mutant, the Δ*sgmT* mutant as well as the Δ*digR*, Δ*sgmT* double mutant had defects in T4P-dependent motility whereas A-motility was unaffected (Fig. 1A). Similarly, to the Δ*digR* mutant, the Δ*sgmT* mutant and the double mutant were unable to undergo fruiting body formation (Fig. S1).

**Fig. 2:**
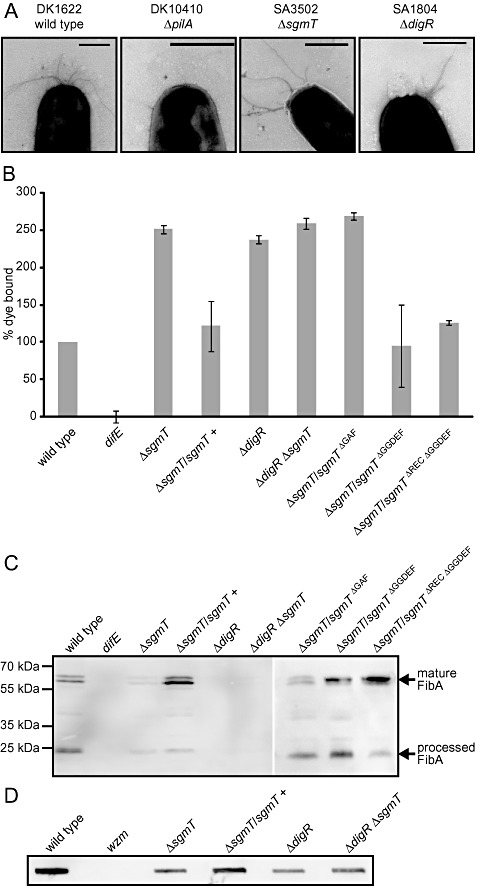
SgmT and DigR are required for correct accumulation of ECM components. A. Δ*sgmT* and Δ*digR* mutants assemble unipolar T4P. Cells from exponentially growing cultures were directly transferred to a grid, stained with 2% (w/v) uranyl acetate and visualized using transmission electron microscopy. Scale bars, 0.5 µm. B. Δ*sgmT* and Δ*digR* mutants accumulate increased amounts of EPS. Cells were treated as in (A). The percentage of dye bound by each sample is indicated relative to WT. Experiments were carried out in triplicate. Standard deviations are indicated. C. Δ*sgmT* and Δ*digR* mutants accumulate less FibA protein. Cells were treated as in (A). Proteins from the same numbers of cells (5 × 10^7^) were loaded per lane, separated by SDS-PAGE and probed with α-FibA antibodies. The positions of mature and processed FibA and molecular size standards are marked. D. Δ*sgmT* and Δ*digR* mutants accumulate less O-antigen. The same number of cells was loaded in each slot and reacted with MAb783, which is specific to the O-antigen portion of LPS.

The Δ*digR* mutant as well as the Δ*sgmT* mutant still assembled unipolar T4P at normal levels. The Δ*pilA* mutant, which is unable to synthesize the T4P subunit, served as a negative control and did not assemble T4P (Fig. 2A). Using the strain SW501, which carries a mutation in *difE* and is defective in synthesis of ECM components (Yang *et al*., 2000), as a negative control, we found that the Δ*digR* mutant, the Δ*sgmT* mutant as well as the Δ*digR*, Δ*sgmT* double mutant accumulated 2.5-fold increased levels of EPS (Fig. 2B). Also, accumulation of the FibA metalloprotease, which is the dominant ECM protein (Behmlander and Dworkin, 1994b), was analysed (Fig. 2C) using SW501 as a negative control. WT accumulates two forms of FibA with apparent sizes of 66 kDa and approximately 25 kDa corresponding to mature FibA and a processed form of FibA (Kearns *et al*., 2002). The Δ*digR* mutant, the Δ*sgmT* mutant as well as the Δ*digR*, Δ*sgmT* double mutant accumulated reduced amounts of both forms. LPS O-antigen by an unknown mechanism is required for T4P-dependent motility (Bowden and Kaplan, 1998). As determined using the strain HK1321, which carries a mutation in *wzm* encoding a subunit of an ABC transporter required for synthesis of the O-antigen (Guo *et al*., 1996), as a negative control, accumulation of LPS O-antigen in the Δ*digR* mutant, the Δ*sgmT* mutant as well as the Δ*digR*, Δ*sgmT* double mutant were slightly reduced (Fig. 2D). Similarly to Δ*digR* mutant, colonies formed by the Δ*sgmT* mutant were slightly less yellow than those of WT. The defects caused by the Δ*sgmT* mutation were corrected by expression of *sgmT* at native levels (Fig. 1C) from the plasmid pTP22 integrated at the phage Mx8 *attB* site. These data confirm that SgmT is important for T4P-dependent motility. Moreover, the similarity of mutant phenotypes suggest that *digR* and *sgmT* act in the same signalling pathway.

### Genetic analysis of SgmT

The deduced SgmT protein consists of 836 amino acids, and contains four domains (Fig. S2A): an N-terminal GAF domain, a histidine kinase domain, which includes the conserved H-Box, with the potential site of autophosphorylation (H336) (Fig. S2B), a receiver domain, which contains all conserved residues (Stock *et al*., 2000) (Fig. S2C) including the potential site of phosphorylation (D615), and a C-terminal GGDEF domain. GAF domains function as sensor domains in many proteins including approximately 9% of histidine protein kinases (Aravind and Ponting, 1997; Gao and Stock, 2009). The GGDEF domain of SgmT contains a degenerate A-site (G^772^GGVF; consensus GGDEF) and an intact I-site (R^763^HPD; consensus RXXD) (Fig. S2D). The Asp and Glu residues of the consensus A-site co-ordinate two magnesium ions involved in catalysis (Wassmann *et al*., 2007) and substitutions to Gly and Val, respectively, as in SgmT have previously been shown to inactivate DGC activity of WspR of *P. fluorescens* (Malone *et al*., 2007). In addition, two positively charged residues (K442 and R446 in PleD; Fig. S2D) that in active enzymes co-ordinate β- and γ-phosphate binding (Wassmann *et al*., 2007) are missing in SgmT, strongly suggesting that the GGDEF domain in SgmT is catalytically inactive but is able to bind c-di-GMP via the intact I-site. SignalP3 (Emanuelsson *et al*., 2007) did not identify a signal peptide in SgmT and TMHMM2 (Krogh *et al*., 2001) did not identify potential trans-membrane spanning regions suggesting that SgmT is localized to the cytoplasm.

To test *in vivo* whether kinase activity is important for SgmT activity, the potential site of autophosphorylation was substituted with Ala (SgmT^H336A^). Moreover, the potential site of phosphorylation (D615) in the receiver domain was substituted to Asn (SgmT^D615N^). SgmT^H336A^ could not substitute for WT SgmT whereas a strain containing SgmT^D615N^ was indistinguishable from WT with respect to T4P-dependent motility and fruiting body formation (Fig. 1B; Fig. S1). To assess the importance of the I-site in the GGDEF domain we generated three substitutions, SgmT^R763A^, SgmT^D766A^ and SgmT^R794A^. R794 is part of a secondary site for c-di-GMP binding (Wassmann *et al*., 2007; Schirmer and Jenal, 2009). Strains containing SgmT^R763A^, SgmT^D766A^ and SgmT^R794A^ were indistinguishable from WT with respect to T4P-dependent motility and fruiting body formation (Fig. 1B; Fig. S1). Finally, we constructed variants of SgmT lacking the GAF domain (SgmT^ΔGAF^), the GGDEF domain (SgmT^ΔGGDEF^) or the receiver-GGDEF domains (SgmT^ΔRECΔGGDEF^) (Fig. S2A). The mutant containing SgmT^ΔGAF^ had motility, developmental and ECM defects (Figs 1B and 2B and C; Fig. S1) similar to the mutants completely lacking SgmT or containing SgmT^H336A^ whereas the two remaining deletions variants of SgmT were similar to WT (Figs 1B and 2B and C; Fig. S1). All mutant SgmT proteins accumulated at levels similar to that of WT SgmT in immunoblots using α-SgmT antibodies (Fig. 1C). Moreover, cell fractionation experiments in which total cell extracts were fractionated into fractions enriched for cytoplasmic, periplasmic or membrane proteins confirmed that SgmT is a cytoplasmic protein (data not shown). In total, these data suggest that SgmT is a cytoplasmic histidine kinase, that the main sensor domain in SgmT is the N-terminal GAF domain, and that the receiver domain and the GGDEF domain are not essential for SgmT activity.

### Global analysis of genes differentially expressed in *ΔsgmT* and *ΔdigR* mutants

DigR contains a C-terminal helix–turn–helix DNA-binding domain of the HTH_Xre type. We hypothesized that SgmT and DigR function together to regulate gene expression. To test this hypothesis, we performed genome-wide expression profiling experiments to determine whether the same set of genes are differentially expressed in the Δ*sgmT* and Δ*digR* mutants. For these experiments we used an *M. xanthus* microarray on which 6687 of the assigned 7380 *M. xanthus* protein-coding genes (90.6%) are represented in triplicate analysable spots of 70-mer single-stranded DNA oligomers (Müller *et al*., 2010). Total RNA was isolated from exponentially growing steady-state cultures of DK1622 (WT), SA3502 (Δ*sgmT*) and SA1804 (Δ*digR*). Microarray analysis was performed using Cy5- (WT) and Cy3-labelled (Δ*sgmT* or Δ*digR*) cDNA that was competitively hybridized to the array in pairwise combinations (Δ*sgmT/*WT and Δ*digR/*WT). Three independent biological experiments were performed and significantly regulated genes were selected by a delta value from a Significance Analysis of Microarrays (SAM) (Tusher *et al*., 2001) analysis where the false discovery rate was 0% in combination with a twofold cut-off criterion.

**Fig. 3:**
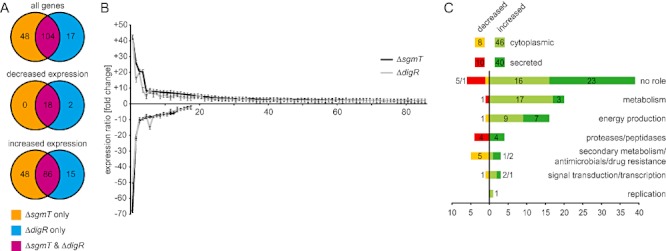
SgmT and DigR co-regulate a large set of genes. A. Genes similarly and differentially affected by lack of SgmT and DigR. In the Venn diagrams, genes similarly affected by lack of SgmT and DigR are indicated in purple and those only affected by lack of SgmT or DigR in orange and blue respectively. B. Genes similarly affected by lack of SgmT and DigR display the same quantitative changes in expression levels. The 86 and 18 genes with increased and decreased expression in the absence of SgmT and DigR were assigned a number from 1 to 86 and 1 to 18, respectively, according to fold increase and decrease in expression levels (Table S1). C. Functional categories of 104 genes similarly affected in Δ*sgmT* and Δ*digR* mutant. In the colour code, the number of proteins predicted to be cytoplasmic or secreted is indicated. Secreted proteins are those predicted to contain a signal peptide type I, a signal peptide type II, a trans-membrane helix, or share homology to an outer membrane protein. All other proteins are classified as cytoplasmic.

Among genes for which data were available for both mutants, 152 and 121 had significantly altered expression levels in the Δ*sgmT* and Δ*digR* mutant respectively (Fig. 3A; Table S1). Among the 121 genes differentially expressed in the Δ*digR* mutant, only 22 were identified in a previous microarray analysis (Overgaard *et al*., 2006). We attribute this discrepancy to differences in microarray technology with the previous microarray being based on a single 275–325 bp PCR fragments per ORF. Importantly, among the genes affected in the Δ*sgmT* and Δ*digR* mutants, 104 were similarly affected in the two mutants and none was oppositely expressed (Table S1). Eighty-six of these 104 genes were expressed at a higher level in the mutants than in WT and 18 genes were expressed at a lower level in the two mutants (Fig. 3A). Moreover, these 104 genes were quantitatively similarly reduced or increased in expression levels in the two mutants (Fig. 3B; Table S1). Generally, genes that were only affected in one of the mutants were two- to threefold increased or decreased in expression levels. To validate the significance of the expression data obtained from the DNA microarrays, quantitative real-time PCR analysis (qRT-PCR) was applied to 13 genes (five genes with reduced expression in the Δ*sgmT* and Δ*digR* mutants, four genes with increased expression in the Δ*sgmT* and Δ*digR* mutants, and four genes expressed at the same levels in WT and the Δ*sgmT* and Δ*digR* mutants). The transcriptional differences determined in the microarray experiments were confirmed by the qRT-PCR analysis (Table S1). The large number of identically affected genes in the Δ*sgmT* and Δ*digR* mutants is in agreement with the notion that SgmT and DigR function in the same pathway.

Fifty-nine of the 104 genes similarly affected in the two mutants encode proteins for which a function can be predicted based on similarity to other proteins and 45 are proteins of unknown function (Fig. 3C). As previously found for the Δ*digR* mutant (Overgaard *et al*., 2006), none of the 59 genes encodes proteins implicated in motility or EPS synthesis and export. Fifty of the 104 genes (48%) are predicted to encode exported proteins (Fig. 3C). Genes with reduced expression in both mutants comprise four predicted secreted proteases including *fibA* and five genes coding for enzymes involved in secondary metabolism including three involved in synthesis of the yellow pigments DKxanthenes that colour *M. xanthus* colonies yellow (Meiser *et al*., 2006) and two potentially involved in synthesis of a type II lantibiotic (Begley *et al*., 2009). The three largest groups of genes of known function with increased expression in both mutants include 20 involved in metabolism, 16 involved in energy production and four proteases, which are all predicted to be secreted (Fig. 3C).

### SgmT phosphotransfers to DigR

**Fig. 4:**
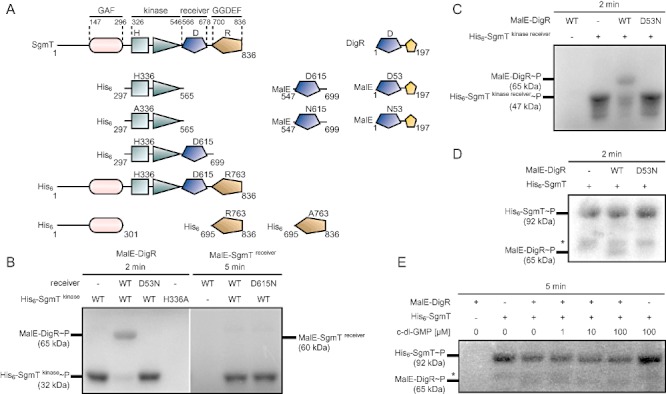
*In vitro* phosphorylation of SgmT and DigR. A. Schematic representation of the domain organization of SgmT and DigR. Constructs used in *in vitro* phosphorylation and c-di-GMP binding experiments are indicated. Numbers indicate the co-ordinates of the constructs with respect to the full-length SgmT. B. Autophosphorylation of SgmT^kinase^ and phosphotransfer to DigR or SgmT^receiver^. SgmT^kinase^ (10 µM final concentration) was incubated in the presence of 1.0 mM [γ-^32^P]-ATP at 30°C for 30 min and then the reaction was either stopped or DigR or SgmT^receiver^ added (10 µM final concentration) for 2 min and 5 min respectively. Samples were separated by SDS-PAGE without prior heating and detected by phosphorimaging. C. Autophosphorylation of SgmT^kinase receiver^ and phosphotransfer to DigR. Samples were treated as in (B). D. Autophosphorylation of full-length SgmT and phosphotransfer to DigR. Samples were treated as described in (B). The asterisk (*) indicates a background band. E. c-di-GMP does not affect full-length SgmT autophosphorylation or phosphotransfer to DigR. Samples were treated as described in (B) except that c-di-GMP was added in the indicated concentrations during autophosphorylation and phosphotransfer. The asterisk (*) indicates a background band.

To test directly whether SgmT and DigR interact, we performed a series of *in vitro* phosphorylation experiments to investigate phosphotransfer within SgmT and between SgmT and DigR (Fig. 4). WT and variants of SgmT were purified from *E. coli* by means of an N-terminal His_6_-tag. DigR and the SgmT receiver domain were purified by means of an N-terminal MalE-tag. In the *in vitro* phosphorylation experiments, SgmT variants were autophosphorylated by incubation for 30 min using [γ-^32^P]-ATP as a phosphodonor. Subsequently, individual receiver proteins in equimolar amounts were added separately. Because cognate histidine kinase/response regulator pairs have a kinetic preference for phosphotransfer *in vitro* (Skerker *et al*., 2005), phosphotransfer reactions were incubated for 2–5 min to avoid unspecific phosphotransfer reactions from occurring.

The histidine kinase domain of SgmT (SgmT^kinase^) was able to autophosphorylate and, as expected, no phosphorylation of the autophosphorylation site mutant SgmT^H336A^ was detected (Fig. 4B). Phosphotransfer to DigR was detected at 2 min as evidenced by the appearance of a radiolabelled band at the molecular mass corresponding to DigR and a decrease in the level of phosphorylated SgmT^kinase^. No phosphotransfer was detected with DigR^D53N^ in which the phosphorylatable D53 residue in the receiver domain (Overgaard *et al*., 2006) was substituted to Asn demonstrating the specificity of the phosphotransfer reaction (Fig. 4B). Specific phosphotransfer was also detected from the SgmT kinase domain containing *in cis* the receiver domain (SgmT^kinase-receiver^) to DigR (Fig. 4C) as well as from full-length SgmT to DigR (Fig. 4D). On the other hand, no phosphotransfer was observed from SgmT^kinase^ to the receiver domain of SgmT (SgmT^receiver^) (Fig. 4B). In combination with the genetic data and transcriptional profiling experiments, these data support the notion that SgmT and DigR comprise a cognate TCS pair of proteins.

### The GGDEF domain binds c-di-GMP and is involved in spatial sequestration of SgmT

**Fig. 5:**
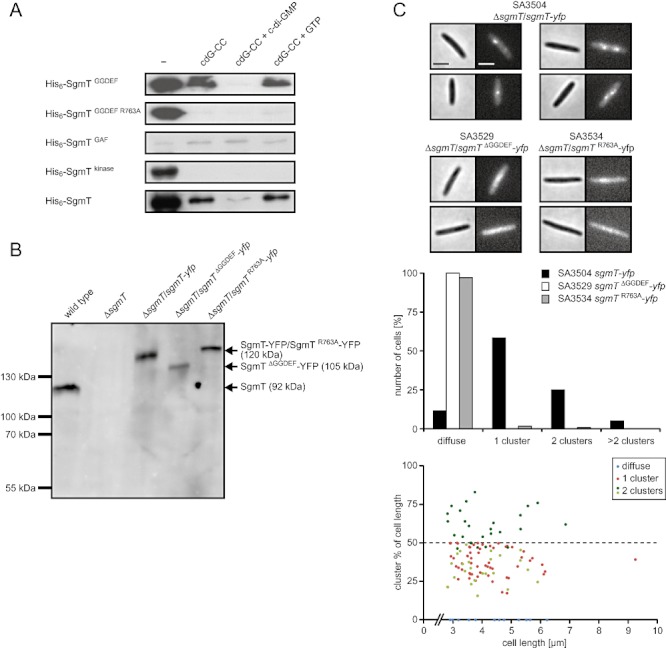
SgmT^GGDEF^ binds c-di-GMP and mediates spatial SgmT sequestration. A. SgmT^GGDEF^ binds c-di-GMP. The indicated proteins (0.5 µM final concentration) were incubated without and with the c-di-GMP capture compound (cdG-CC, 5 µM final concentration) and in the absence or presence of c-di-GMP or GTP (500 µM final concentration) as indicated, isolated, separated by SDS-PAGE and identified by immunoblotting using α-SgmT antibodies. The lane labelled ‘–’ contains the total amount of protein added to each reaction. See Fig. 4A for details of constructs used. B. Immunoblot analysis of the accumulation of SgmT–YFP variants. Total cell lysates from exponentially growing cells were separated by SDS-PAGE, and probed with α-SgmT antibodies. Proteins of the same numbers of cells (5 × 10^7^) were loaded per lane. Positions of SgmT variants, and molecular size markers are indicated. C. c-di-GMP binding to SgmT^GGDEF^ sequesters SgmT. Cells were transferred from exponentially growing cultures to a thin 1.0% agar pad on a microscope slide, and imaged by fluorescence and phase-contrast microscopy. Left and right panels show phase-contrast and fluorescence images respectively. The histogram shows the distribution of cells with diffuse localization and one, two or more clusters. The scatter diagram shows cluster localization for SgmT–YFP as a function of cell length. Cells with diffuse localization are indicated by blue symbols at 0% and pairs of clusters in cells with two clusters are indicated by light and dark green symbols. The stippled line indicates midcell. Scale bar, 2 µm.

A trifunctional compound composed of a c-di-GMP moiety, a photo-activatable group to covalently cross-link bound proteins, and a biotin to pull out the captured protein (c-di-GMP-capture compound, cdG-CC), was used to test whether SgmT specifically binds c-di-GMP. Purified SgmT variants were tested for capturing with 5 µM cdG-CC. Following incubation and cross-linking, the complex was isolated using streptavidin coated magnetic beads, and analysed by SDS-PAGE and immunoblots using α-SgmT antibodies. As shown in Fig. 5A, the isolated SgmT GGDEF domain was readily captured. This binding was outcompeted by c-di-GMP (500 µM final concentration) but not by GTP (500 µM final concentration). As expected, the GGDEF domain variant with the substitution of the conserved Arg residue in the I-site (SgmT^GGDEF R763A^) was unable to bind the cdG-CC. The isolated GAF domain is only poorly recognized by the α-SgmT antibodies (Fig. 5A, lane 1); however, the amount of protein captured by the probe was similar in the absence and presence of excess c-di-GMP or GTP suggesting that the GAF domain does not bind c-di-GMP. The isolated SgmT kinase domain did not bind c-di-GMP; however, full-length SgmT specifically bound c-di-GMP.

Having established that the GGDEF domain of SgmT alone as well as in the context of the full-length protein is able to bind c-di-GMP *in vitro*, we next asked whether full-length SgmT autophosphorylation or phosphotransfer from full-length SgmT to DigR is regulated by c-di-GMP. As shown in Fig. 4E, neither autophosphorylation of full-length SgmT nor phosphotransfer to DigR was significantly changed by the addition of c-di-GMP.

Several proteins involved in c-di-GMP metabolism and regulation localize to specific subcellular regions (Hengge, 2009). To observe the localization of SgmT, we constructed an *sgmT–yfp* fusion expressed at native levels (Fig. 5B). The SgmT–YFP fusion protein was functional as Δ*sgmT* cells expressing the fusion displayed WT motility (Fig. S3A). In cells transferred from exponentially growing cultures to an agar pad on a microscope slide, SgmT–YFP localized to a single cluster along the cell length in 58% of cells (Fig. 5C) (from here on referred to as the cell body cluster) with the mean localization of the cluster at 37.7 ± 8.8% of the cell length (*n* = 100). Twenty-five per cent of cells contained two clusters centred around midcell at 33.3 ± 9.1% and 61.0 ± 10.4% of the cell length. The remaining 17% contained either more than two clusters (5%) or SgmT–YFP localized diffusely throughout the cytoplasm (12%). Time-lapse fluorescence microscopy demonstrated that the clusters were stationary and not dynamic (Fig. S3B). SgmT^ΔGGDEF^–YFP as well as the I-site mutant SgmT^R763A^–YFP did not localize to clusters but rather localized diffusely throughout the cytoplasm (Fig. 5C) suggesting that c-di-GMP binding to the I-site mediates SgmT localization to the cell body clusters.

These observations suggested that SgmT may respond to varying levels of c-di-GMP by I-site-mediated sequestration to the cell body clusters. To demonstrate that SgmT localizes in response to varying levels of c-di-GMP, we attempted to express Strep-tagged versions of two highly active DGCs [VCA0956 from *Vibrio cholerae* (Tischler and Camilli, 2004) and PleD* from *C. crescentus* (Paul *et al*., 2004)] and one PDE [YhjH from *Salmonella typhimurium* (Simm *et al*., 2004)] in *M. xanthus* in order to increase or decrease, respectively, the intracellular c-di-GMP concentration. The corresponding genes were expressed from the constitutively active *pilA* promoter in plasmids integrated at the Mx8 *attB* site. However, all three proteins were undetectable in *M. xanthus* suggesting that they are rapidly degraded, thus, precluding a detailed analysis of varying c-di-GMP levels on SgmT localization.

To further understand the function of the GGDEF domain, whole-genome expression profiling experiments were performed in which the Δ*sgmT* mutant complemented with *sgmT*^+^ (SA3503) and the Δ*sgmT* mutant complemented with *sgmT*^ΔGGDEF^ (SA3511) were compared. Briefly, total RNA was isolated from exponentially growing cultures of SA3503 and SA3511 and analysed using the method described above. Interestingly, we did not observe significant differences in the expression profiles of the two strains under the conditions tested.

### Identification of the DigR DNA binding site

**Fig. 6:**
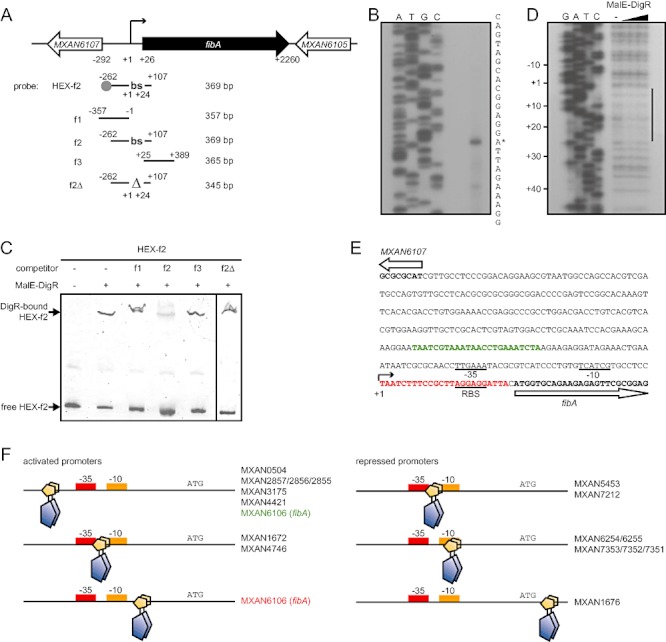
DigR binds specifically to the *fibA* promoter. A. Schematic overview of the *fibA* gene and flanking genes. Co-ordinates are relative to the transcription start site (+1). DNA fragments used in EMSA are shown. B. Determination of the transcription start site of *fibA*. The ^32^P-5′-labelled primer f2-rev was hybridized to total RNA from exponentially growing WT DK1622 followed by reverse transcription. Lanes 1–4 are the A, T, G and C sequencing reactions. Lane 5, the reverse transcriptase reaction. The DNA sequence shown to the right represents the sequence of non-coding strand DNA. The transcription start site is indicated as A*. C. Electrophoretic mobility shift assays of DigR binding to the *fibA* promoter. MalE-DigR (4 µM final concentration) was mixed with the HEX-labelled f2 probe (2.5 nM final concentration) in the presence or absence of 150-fold molar excess of competitor DNA fragments as indicated. Free HEX-f2 and DigR bound HEX-f2 are indicated. D. DNase I footprinting analysis of DigR binding to the *fibA* promoter. MalE-DigR was mixed with a DNA fragment extending from −262 to +107 ^32^P-labelled at the 5′-end of the lower strand. Final concentrations of DigR in the four binding reactions were 0, 8, 16 and 24 µM respectively. The region protected from cleavage by DNase I is shown by a vertical line. Sequence ladders generated by dideoxy sequencing using the ^32^P-end-labelled primer f2-rev (lanes 1–4) were used to locate precisely the protected region. E. DNA sequence of the *fibA* promoter. The transcription start site is indicated by the kinked arrow. The start of the structural *fibA* gene is indicated. The −10 and −35 regions of the promoter and the Shine–Dalgarno sequence (RBS) are underlined. The DigR binding site identified by EMSA and DNase I footprinting experiments is indicated red; the degenerate DigR binding site is indicated in green. F. Schematic overview of DigR binding sites of activated (left) and repressed (right) promoters. Note that among activated promoters, MXAN2857 is included. This gene is likely in an operon with MXAN2856 and MXAN2855 but is not significantly affected in expression in both the Δ*sgmT* and Δ*digR* mutants. Among the repressed promoters, MXAN7353 is less than 4.0-fold reduced in expression in the Δ*digR* mutant. It is included here because it is likely in an operon with MXAN7351 and MXAN7352 (Tables S3 and S4). Suggested promoter elements (−35 and −10) are displayed as red and orange boxes respectively. Translation start is displayed as ATG, suggested DigR binding sites are indicated by the connected blue and yellow pentagons. Colour code for DigR binding sites in the *fibA* promoter is as in (E).

To determine which genes are directly regulated by the SgmT/DigR TCS, we initially focused on *fibA* because it encodes a protein of known function and its transcription is strongly downregulated in the absence of SgmT and DigR. To map the *fibA* transcription start site, primer extension experiments were performed using total RNA isolated from exponentially growing *M. xanthus* cells. In these experiments, a single extension product was evident (Fig. 6B). This extension product mapped the transcription start site to a T 25 bp upstream from the *fibA* ATG start codon (Fig. 6B). Inspection of the DNA sequence upstream of the *fibA* transcriptional start site revealed the presence of the hexanucleotide T^−36^TGAAA, which is a good match to the consensus −35 sequence of bacterial σ^70^ promoters (TTGACA) (van Hijum *et al*., 2009) (Fig. 6E). The best match to the −10 consensus sequence of bacterial σ^70^ promoters is T^−13^CATCG, which only poorly matches the consensus (TATAAT). The poor match to the −10 sequence may explain the low promoter activity in a Δ*digR* strain and why the *fibA* promoter depends on DigR for high activity.

To identify potential DigR binding sites in the *fibA* promoter region, we carried out electrophoretic mobility gel shift assays (EMSA). Several response regulators autophosphorylate using low-molecular-weight phosphodonors such as acetylphosphate (Lukat *et al*., 1992). However, we were unable to detect autophosphorylation of DigR in the presence of ^32^P-labelled acetylphosphate. Therefore, non-phosphorylated MalE-DigR was used in the *in vitro* DNA binding experiments. DigR bound to a hexachlorofluorescein-(HEX) labelled *fibA* promoter fragment (HEX-f2) spanning from −262 to +107 (Fig. 6A and C). Binding to HEX-f2 was outcompeted by 150-fold molar excess of unlabelled f2 fragment but not by similar excess of f1 fragment (−357 to +1) or by f3 fragment (+25 to +389) (Fig. 6A and C) demonstrating that binding of DigR to the HEX-f2 fragment is specific and suggesting that a DigR binding site localizes between +1 and +24. Consistently, the f2Δ fragment, which extends from −262 to +107 but lacks the +1 to +24 sequence, did not outcompete DigR binding to the HEX-f2 fragment (Fig. 6A and C).

To confirm the DigR binding site identified in the EMSA, we carried out DNase I footprinting experiments with the f2 fragment ^32^P-end-labelled at the 5′-end of the lower strand. As shown in Fig. 6D, DigR bound specifically to the region spanning from +1 to +24 relative to the transcription start site. This region contains a palindrome (5′-TAATCTTTCCGCTTAGGAGGATTA; palindrome indicated by underlining) suggesting that DigR binds as a dimer.

### Mapping of the SgmT/DigR regulon

To identify other genes likely to be directly regulated by DigR, we searched the promoter sequences of genes similarly altered in expression in the absence of DigR and SgmT in the microarray experiment for the outermost part of the palindrome in the DigR binding site identified in the *fibA* promoter (TAATC-N14-GATTA) allowing up to four mismatches. In cases where a gene with altered expression was thought to be part of an operon, the promoter region of the first gene in the operon was searched. It should be noted that this type of analysis is challenging because only few *M. xanthus* transcriptional start sites and operons have been mapped. Moreover, among the genes with altered expression in the absence of SgmT and DigR at least two code for transcriptional regulators [MXAN5125 (*mrpC*) and MXAN1093] and, thus, also contribute to changes in gene expression in the Δ*sgmT* and the Δ*digR* mutants that are only indirectly caused by lack of SgmT or DigR.

Using this approach, eight of the 18 genes with decreased expression in both the Δ*sgmT* and Δ*digR* mutants were found to contain a variant of the DigR binding site (Fig. 6F; Tables S1–S4). Similarly, among the 17 genes displaying a more than 4.0-fold increased expression in the Δ*sgmT* and the Δ*digR* mutants, eight contained a variant of the DigR binding site either in their promoter region or in the promoter region of the first gene of an operon.

DigR binding sites in promoters activated by DigR were found at different positions varying from upstream a −35 sequence, between the −35 and −10 sequences, and immediately downstream of the +1 sequence (the *fibA* promoter) (Fig. 6F). Similarly, in promoters repressed by DigR, the binding sites were identified in different locations within promoter regions (Fig. 6F). It should be noted that non-phosphorylated DigR was used to determine the DigR binding site in the *fibA* promoter. It has been described for other DNA binding response regulators that phosphorylation stimulates binding to low-affinity binding sites (e.g. Dahl *et al*., 1997) without changing the DNA sequence recognized. The *fibA* promoter also contains a variant of the palindromic DigR binding site upstream of the −35 sequence (5′-TAATCGTAAATAACCTGAAATCTA), which essentially consists of one half of the palindrome in the mapped binding site. Binding to this site was detected neither in EMSA (Fig. 1C) nor in DNase I footprinting experiments suggesting that it is a low-affinity binding site for DigR and only binds DigR in the phosphorylated form.

## Discussion

SgmT is an orphan hybrid histidine kinase with a receiver domain C-terminal to the kinase domain. *In vitro* phosphorylation experiments provided direct evidence for autophosphorylation of the SgmT kinase domain on H336; however, phosphotransfer was not detected to the isolated SgmT receiver domain. In contrast, efficient phosphotransfer was observed to D53 in the DigR receiver domain. Thus, SgmT^kinase^ has a kinetic preference for DigR over isolated SgmT^receiver^. Consistently, the two receiver domains only share 25% identity and 43% similarity and the amino acid residues suggested to be involved in specific contact to the kinase domain (Capra *et al*., 2010) are highly divergent (Fig. S2B). In the case of hybrid kinases, an *in vitro* kinetic preference for a separate response regulator over its own receiver domain using isolated domains can be outperformed in the context of the full-length kinase protein by the spatial proximity of the *in cis* receiver (Wegener-Feldbrügge and Søgaard-Andersen, 2009). However, this does not seem to be the case for SgmT because phosphotransfer to DigR was also observed with a SgmT variant consisting of the kinase and receiver domains as well with full-length SgmT. Hybrid HPKs can be part of phosphorelays consisting of a histidine protein kinase, a response regulator, a phosphotransmitter (Hpt) and a second response regulator. These systems function as simple TCS except that phosphotransfer occurs in three sequential steps (Appleby *et al*., 1996). To our knowledge, it has not been observed that a kinase in a phosphorelay engages directly in phosphotransfer to the response regulator in the last response regulator in a phosphorelay. Rather transfer of the phosphoryl group to the last response regulator strictly depends on an Hpt domain (Takeda *et al*., 2001; Biondi *et al*., 2006; Hsu *et al*., 2008). Therefore, we consider it unlikely that SgmT and DigR are part of a phosphorelay. The receiver domain is conserved in SgmT homologues in other species (Fig. S4) suggesting a selective pressure to maintain this domain. Yet, our genetic and biochemical data suggest that under the conditions tested, this domain is not essential for SgmT function. On the basis of the genetic and biochemical data, we suggest that the orphan SgmT histidine kinase and the orphan DNA binding response regulator DigR constitute a cognate pair of TCS proteins with SgmT directly transferring a phosphate group to DigR.

Using a combination of EMSA and DNase I footprinting, we identified a DigR binding site in the *fibA* promoter. This binding site is palindromic suggesting that DigR binds as a dimer. Eight of 18 genes with decreased expression in the absence of SgmT and DigR in whole-genome expression profiling experiments contain this sequence or variants in their promoter region (or if part of a potential operon, in the promoter region of the operon). Similarly, among the 17 genes displaying a more than 4.0-fold increased expression in the absence of DigR, seven contain a variant of the DigR binding site either in their promoter region (or, if part of a potential operon, in the promoter region of the operon). The observation that promoters with decreased expression as well as promoters with increased expression in the absence of SgmT and DigR both contain a DigR binding site suggests that DigR functions as an activator as well as a repressor of transcription. The DigR operator locations are in agreement with operator locations in *E. coli* promoters for activators as well as repressors except that binding of an activator as observed in the *fibA* promoter in the proximal promoter region is unusual (Madan Babu and Teichmann, 2003; Cox *et al*., 2007; van Hijum *et al*., 2009).

The eight genes with decreased expression in the absence of SgmT and DigR and which contain a DigR binding site code for the secreted protease FibA, an outer membrane TonB-dependent receptor, two enzymes involved in lantibiotic synthesis, and four hypothetical proteins predicted to be secreted and only encoded by the *M. xanthus* genome (Huntley *et al*., 2011). Six of the seven genes with increased expression in the absence of SgmT and DigR and which contain a DigR binding site code for secreted or non-secreted hypothetical proteins with a mostly broad distribution phylogenetically and the remaining gene code for an oxidase. The *sgmT* and *digR* mutants were originally identified based on their defect in T4P-dependent motility. Because none of the genes affected in their expression in the absence of SgmT and DigR are known to cause a defect in T4P-dependent motility or are involved in EPS biosynthesis and export, we speculate that the SgmT/DigR TCS activates genes coding for proteins secreted to the ECM, i.e. FibA and four hypothetical proteins, and for enzymes involved in secondary metabolism. In their absence, cells as a compensatory response secrete increased amounts of EPS. As previously proposed for the Δ*digR* mutant (Overgaard *et al*., 2006), we propose that the defect in T4P-dependent motility in the Δ*sgmT* mutant is caused by abnormal accumulation of EPS. It was previously noted that the ECM is enriched for proteins of unknown function (Curtis *et al*., 2007). Consistently, several of the genes suggested to be directly regulated by SgmT/DigR code for *M. xanthus*-specific proteins of unknown function and likely secreted to the ECM. Future research will address the function of these proteins.

SgmT contains an N-terminal GAF domain, which is essential for activity *in vivo*. GAF domains are found in a multitude of proteins and are typically involved in regulating the activity of an adjacent domain with enzyme activity including the activity of histidine kinases (Gao and Stock, 2009). GAF domains bind cyclic nucleotides [cAMP and cGMP (Heikaus *et al*., 2009)], NO in the context of a non-haem iron centre (Tucker *et al*., 2008) and tetrapyrroles (Tilman, 2004). We do not know the ligand that binds to the SgmT GAF domain. We speculate that binding of this ligand leads to conformational changes in SgmT resulting in activation of histidine kinase activity and autophosphorylation. The sequence of the A-site in the GGDEF domain is incompatible with DGC activity (Malone *et al*., 2007). However, the GGDEF domain contains an intact I-site. *In vitro* analyses demonstrated that this domain alone as well as in the context of full-length SgmT binds c-di-GMP and this binding depends on an intact I-site. Moreover, in the absence of the GGDEF domain or in a mutant containing a mutant I-site unable to bind c-di-GMP *in vitro*, SgmT localizes diffusely throughout the cytoplasm whereas WT protein is sequestered in one or more clusters localized along the cell length. These observations suggest that the GGDEF domain functions as a receptor for c-di-GMP and that one function of c-di-GMP binding to the I-site *in vivo* is the spatial sequestration of SgmT. This function is similar to that described for the GGDEF domain in the *C. crescentus* response regulator PopA, in which polar sequestration depends on c-di-GMP binding to an intact I-site in the GGDEF domain with a degenerate A-site (Duerig *et al*., 2009). In conclusion, the data presented suggest that the GAF domain is the major sensor domain regulating SgmT kinase activity and that the GGDEF domain is a c-di-GMP receptor, a function of which is to sequester SgmT to the cell body cluster upon c-di-GMP binding. To our knowledge SgmT is the first histidine kinase that functions as a receptor for c-di-GMP.

What is the function of the c-di-GMP-dependent sequestration of SgmT? Among species with sequenced genomes, SgmT homologues are found in six myxobacteria. In these SgmT homologues, the four domain structure including the C-terminal GGDEF domain with a degenerate A-site and an intact I-site is conserved (Fig. S4) suggesting a strong selective pressure to maintain this domain in SgmT. Yet, no phenotypic or gene expression differences were evident between WT and a mutant lacking the GGDEF domain. Similarly, in *in vitro* phosphorylation experiments c-di-GMP had an effect neither on SgmT autophosphorylation nor on SgmT phosphotransfer to DigR. The only function currently assigned to the GGDEF domain is the spatial sequestration of SgmT upon c-di-GMP binding. Localization of TCS proteins to specific subcellular regions is important for minimizing cross-talk between signalling proteins (Scott *et al*., 2010), enabling the asymmetric inheritance of cell fate determinants after cell division (Wheeler and Shapiro, 1999), sequestration of a kinase from an inhibitor (Tsokos *et al*., 2011), signal amplification (Wadhams and Armitage, 2004) and spatial confinement of a signalling process (Abel *et al*., 2011). Our data suggest that under the conditions tested, SgmT lacking the GGDEF domain displayed neither more nor less activity than WT SgmT. We speculate that under certain conditions the c-di-GMP-dependent spatial sequestration of SgmT may serve to insulate SgmT and/or DigR from cross-talk from other signalling pathways.

It is an open question how the same second messenger produced by different GGDEF domain proteins can elicit different responses. It has been proposed (Hengge, 2009) that different c-di-GMP-dependent signal transduction pathways are insulated from each other either by mechanisms involving the temporal separation of different pathways, i.e. the components of these pathways are present in a cell under different conditions, or by mechanisms involving the spatial separation of different pathways, i.e. the components of different pathways are present in a cell at the same time but insulated from each other by localizing to different subcellular regions. A prediction from the model for the function of the GGDEF domain in SgmT is the existence of a catalytically active GGDEF domain protein(s) that function to recruit SgmT to the cell body clusters. The *M. xanthus* genome encodes 24 proteins involved in c-di-GMP metabolism and regulation including 17 proteins with a GGDEF domain, two with an EAL domain and five with an HD-GYP domain (Table S4). Among these 24 proteins only SgmT and ActA (Gronewold and Kaiser, 2001) have been analysed. ActA contains an N-terminal receiver domain followed by a GGDEF domain with a degenerate A-site and an intact I-site. ActA has no effect on motility and is required for fruiting body formation in response to starvation (Gronewold and Kaiser, 2001). Among the remaining 15 proteins with GGDEF domains, 11 are predicted to have an intact A-site based on sequence analyses (Table S4). Among the latter 11 proteins, we are currently systematically trying to identify those involved in the spatial sequestration of SgmT.

## Experimental procedures

### Strains, cell growth and development

*Myxococcus xanthus* cells were grown in CTT medium or on CTT agar plates (Hodgkin and Kaiser, 1977). All *M. xanthus* strains are derivatives of the wild-type DK1622 (Kaiser, 1979). *M. xanthus* strains and plasmids used in this work are listed in Tables 1 and 2. The in-frame deletion of *sgmT* was generated as described (Shi *et al*., 2008) using the plasmid pTP11 and contains a deletion extending from codon 54 (CTG) to codon 823 (AAG) in *sgmT*. Kanamycin and oxytetracycline were used for selective growth at concentrations of 40 or 10 µg ml^−1^ respectively. *E. coli* strains were grown in LB broth in the presence of relevant antibiotics (Sambrook and Russell, 2001). All plasmids were propagated in *E. coli* strain Mach1 [Δ*recA*1398 *endA*1 *tonA*Φ80Δ*lacM*15 Δ*lacX*74 *hsdR*(r_K_^-^ m_K_^+^)] unless otherwise stated. For motility assays, cells were grown in CTT broth to a density of 7 × 10^8^ cells ml^−1^, harvested and resuspended in 1% CTT to a calculated density of 7 × 10^9^ cells ml^−1^. Five-microlitre aliquots of cells were placed on 0.5% and 1.5% agar supplemented with 0.5% CTT and incubated at 32°C. After 24 h, colony edges were observed using a Leica MZ8 stereomicroscope or in a Leica IMB/E inverted microscope and visualized using Leica DFC280 and DFC350FX CCD cameras respectively. Fruiting body formation was monitored on TPM agar (10 mM Tris-HCl pH 7.6, 1 mM K_2_HPO_4_/KH_2_PO_4_ pH 7.6, 8 mM MgSO_4_) as described (Søgaard-Andersen *et al*., 1996).

**Table 1:** *M. xanthus* strains used in this work

*M. xanthus* strains	Genotype^a^	Reference
DK1622	Wild type	Kaiser (1979)
DK1217	*cglB2*	Hodgkin and Kaiser (1979)
DK1300	*sglG1*	Hodgkin and Kaiser (1979)
DK10410	Δ*pilA*	Wu and Kaiser (1997)
DK11063	*fruA::Tn5 lacΩ7540*	Søgaard-Andersen *et al*. (1996)
SW501	*difE*::kan^R^	Yang *et al*. (1998)
HK1321	*wzm::*kan^R^	Bowden and Kaplan (1998)
SA1804	Δ*digR*	Overgaard *et al*. (2006)
SA3502	Δ*sgmT*	This study
SA3503	Δ*sgmT/*P*_nat_-sgmT* (pTP22), tet^R^	This study
SA3506	Δ*sgmT/*P*_nat_-sgmT*^H336A^ (pTP24), tet^R^	This study
SA3507	Δ*sgmT/*P*_nat_-sgmT*^D615N^ (pTP25), tet^R^	This study
SA3508	Δ*sgmT/*P*_nat_-sgmT*^R763A^ (pTP26), tet^R^	This study
SA3509	Δ*sgmT/*P*_nat_-sgmT*^D766A^ (pTP27), tet^R^	This study
SA3510	Δ*sgmT/*P*_nat_-sgmT*^R794A^ (pTP28), tet^R^	This study
SA3511	Δ*sgmT/*P*_nat_-sgmT*^ΔGGDEF^ (pTP40), tet^R^	This study
SA3512	Δ*sgmT/*P*_nat_-sgmT*^ΔGAF^ (pTP39), tet^R^	This study
SA3513	Δ*sgmT/*P*_nat_-sgmT*^ΔREC ΔGGDEF^ (pTP29), tet^R^	This study
SA3515	Δ*digR*Δ*sgmT*	This study
SA3504	Δ*sgmT/*P*_nat_-sgmT–yfp* (pTP41), tet^R^	This study
SA3529	Δ*sgmT/*P*_nat_-sgmT*^ΔGGDEF^*–yfp* (pTP44), tet^R^	This study
SA3534	Δ*sgmT/*P*_nat_-sgmT*^R763A^*–yfp* (pTP46), tet^R^	This study

a. Plasmids in brackets were integrated at the Mx8 *attB* attachment site by site-specific recombination.

**Table 2:** Plasmids used in this work

Plasmids	Description^a^	Reference
pBJ114	kan^R^, *galK*	Julien *et al*. (2000)
pSWU30	tet^R^	Wu and Kaiser (1997)
pET28a(+)	kan^R^, expression vector	Novagen
pMAL-c2x	ap^R^, expression vector	New England Biolabs
pTP11	pBJ114, KpnI-flanking regions of *sgmT* (*MXAN4640*)-XbaI	This study
pTP22	pSWU30, XbaI-P_nat_+*sgmT*-EcoRI	This study
pTP24	pSWU30, XbaI-P_nat_+*sgmT*^H336A^-EcoRI	This study
pTP25	pSWU30, XbaI-P_nat_+*sgmT*^D615N^-EcoRI	This study
pTP26	pSWU30, XbaI-P_nat_+*sgmT*^R763A^-EcoRI	This study
pTP27	pSWU30, XbaI-P_nat_+*sgmT*^D766A^-EcoRI	This study
pTP28	pSWU30, XbaI-P_nat_+*sgmT*^R794A^-EcoRI	This study
pTP39	pSWU30, XbaI-P_nat_+*sgmT*^ΔGAF^-EcoRI	This study
pTP40	pSWU30, XbaI-P_nat_+*sgmT*^ΔGGDEF^-EcoRI	This study
pTP29	pSWU30, XbaI-P_nat_+*sgmT*^ΔREC ΔGGDEF^-EcoRI	This study
pTP41	pSWU30, XbaI-P_nat_+*sgmT–yfp*-EcoRI	This study
pTP44	pSWU30, XbaI-P_nat_+*sgmT*^ΔGGDEF^*–yfp*-EcoRI	This study
pTP46	pSWU30, XbaI-P_nat_+*sgmT*^R763A^*–yfp*-EcoRI	This study
pTP33	pET28a(+), NdeI-*His6*-*sgmT*-EcoRI	This study
pTP51	pET28a(+), NdeI-*His6-sgmT*^kinase^-EcoRI	This study
pTP52	pET28a(+), NdeI-*His6-sgmT*^kinase H336A^-EcoRI	This study
pTP53	pET28a(+), NdeI-*His6-sgmT*^kinase receiver^-EcoRI	This study
pTP55	pMAL-c2x, EcoRI-*MalE*-*sgmT*^receiver^-BamHI	This study
pTP56	pMAL-c2x, EcoRI-*MalE-sgmT*^receiver D615N^-BamHI	This study
pTP57	pMAL-c2x, EcoRI-*MalE-digR*-BamHI	This study
pTP58	pMAL-c2x, EcoRI-*MalE-digR*^D53N^-BamHI	This study
pTP59	pET28a(+), NdeI-*His6-sgmT*^GAF^-EcoRI	This study
pTP60	pET28a(+), NdeI-*His6*-*sgmT*^GGDEF^-EcoRI	This study
pTP62	pET28a(+), NdeI-*His6*-*sgmT*^GGDEF R763A^-EcoRI	This study

a. Restriction sites included were used for cloning in the indicated cloning vector.

### Tryphan blue binding assay

To quantify binding of Tryphan blue, a liquid binding assay was adapted from Black and Yang (2004) except that 5 × 10^8^ cells from exponential cultures were harvested, washed and resuspended in 900 µl of 10 mM MOPS pH 7.6, 1 mM MgSO_4_ buffer.

### Immunoblot analysis and cell fractionation

Immunoblots were carried out as described (Sambrook and Russell, 2001) using rabbit α-SgmT antibodies and α-FibA monoclonal antibody MAb2105 (Kearns *et al*., 2002). Secondary antibodies were either horseradish-conjugated goat anti-rabbit immunoglobulin G (SgmT) or horseradish-conjugated rabbit anti-mouse immunoglobulin G (FibA). O-antigen was quantified in slotblots of whole cells as described using the monoclonal MAb783 antibody (Guo *et al*., 1996). Blots were developed using Luminata crescendo Western HRP Substrate (Millipore). To generate rabbit, polyclonal α-SgmT antibodies, His_6_-SgmT was used to immunize rabbits using standard procedures (Sambrook and Russell, 2001). Fractionation of total cell extracts into fractions enriched for cytoplasmic, periplasmic or membrane proteins were carried out as described (Lobedanz and Søgaard-Andersen, 2003) using cells growing exponentially in CTT.

### Transmission electron microscopy

Cells growing exponentially in CTT were analysed as described (Jakovljevic *et al*., 2008). Transmission electron microscopy was performed on a Philips EM 301 electron microscope at calibrated magnifications.

### DNA microarray analysis and qRT-PCR analysis

DNA-free total RNA was isolated from cells growing exponentially in CTT medium using the hot-phenol method as described (Overgaard *et al*., 2006). cDNA synthesis and fluorescent labelling of cDNA with Cy3 or Cy5 were done as described (Shi *et al*., 2008). Probes were hybridized to the microarray as described (Müller *et al*., 2010). qRT-PCR was performed in a 25 µl reaction volume using SYBR green PCR master mix (Applied Biosystems) and 0.1 µM primers specific to the target gene in a 7300 Real Time PCR System (Applied Biosystems). To exclude chromosomal DNA contamination, a control reaction for each sample was performed using equivalent amount of RNA solution as a template. Each reaction was performed in triplicate. Relative expression levels were calculated using the comparative Ct method.

### Microarray data accession number

The microarray data discussed in this publication have been deposited in the NCBIs Gene Expression Omnibus (http://www.ncbi.nlm.nih.gov/geo/) and are accessible through Gene Expression Omnibus Series Accession No. GSE34408.

### *In vitro* phosphotransfer

*In vitro* phosphorylation of His_6_-SgmT proteins was carried out in TGMNK buffer [50 mM Tris-HCl pH 8.0, 10% (v/v) glycerol, 10 mM MgCl_2_, 150 mM NaCl, 50 mM KCl] at 30°C. SgmT variants (10 µM or 5 µM) were incubated with 1.0 mM [γ-^32^P]-ATP (44.4 TBq mmol^−1^; Hartmann Analytic) for 30 min. Subsequently, receiver domain proteins were added for 2 or 5 min. Aliquots of 10 µl were quenched in 5 µl of 3× SDS/EDTA loading buffer [7.5% (w/v) SDS, 90 mM EDTA, 37.5 mM Tris-HCl (pH 6.8), 37.5% glycerol (v/v), 0.3 M DTT] and separated by SDS-PAGE at 20°C without prior heating (Rasmussen *et al*., 2006). Subsequently, the gel was exposed on a phosphorimager screen (GE Healthcare) and scanned on a Storm phosphorimager (GE Healthcare).

### c-di-GMP binding

The capture experiments with cdG-CC (caprotec bioanalytics GmbH) were essentially performed as described (J. Nesper *et al*., in preparation). Briefly, proteins (0.5 µM) and cdG-CC (5 µM) were incubated in 100 µl of 20 mM HEPES pH 7.5, 50 mM KAc, 10 mM MgAc, 10% glycerol for 1 h at 4°C in the dark. UV cross-linking was performed for 4 min at 310 nm at −3°C using a caproBox (caprotec). After addition of 50 µl of streptavidin-coated magnetic beads (Dynabeads MyOne Streptavidin C1, Invitrogen) and 25 µl of 5× wash buffer (250 mM Tris pH 7.5, 5 M NaCl, 0.1% n-octyl-β-glucopyranoside), samples were incubated for 1 h at 4°C. Beads were washed 6× in 1× wash buffer, isolated and boiled in 1× SDS loading buffer for 10 min. Proteins were separated using SDS-PAGE and bound proteins identified by immunoblotting using α-SgmT antibodies. For competition assays, proteins and competitor (c-di-GMP or GTP, 500 µM final concentration) were incubated at 4°C for 30 min before adding cdG-CC.

### Microscopy

For fluorescence microscopy, *M. xanthus* cells were grown in suspension as described, placed on a thin 1.0% agar-pad buffered with A50 starvation buffer (10 mM MOPS pH 7.2, 1.0 mM CaCl_2_, 1.0 mM MgCl_2_, 50 mM NaCl) on a glass slide, immediately covered with a coverslip and imaged using a Leica DM6000B microscope with a Leica Plan Apo 100×/NA1.40 phase-contrast oil objective and a Cascade II:1024 camera (Photometrics). For time-lapse recordings, cells were treated as described and imaged at 30 s intervals for 15 min. A Leica YFP filter (excitation range 490–510 nm, emission range 520–550 nm) was used for visualizing YFP-tagged SgmT. Images were recorded with Image Pro 6.2 (Media Cybernetics) and processed with Metamorph 7.7.5.0 software (Molecular Devices). Processed images were arranged in Adobe Photoshop CS2.

### Protein purification

For expression and purification of His_6_-tagged or MalE-tagged proteins, constructs were constructed in pET28a(+) (Novagen) or pMAL-c2X (New England Biolabs) respectively (Table 2). All proteins were expressed in *E. coli* Rosetta 2(DE3) [F^-^*ompT hsdS*_B_(r_B_^-^ m_B_^-^) *gal dcm* (DE3) pRARE2] at 18°C. His_6_-tagged proteins were purified using Ni-NTA affinity purification. Briefly, cells were resuspended in buffer A (50 mM Tris-HCl, 150 mM NaCl, 10 mM imidazole, 1 mM DTT, 10% glycerol pH 8) and lysed by sonication. After centrifugation, lysates were loaded on Ni-NTA agarose column (Qiagen) and washed with 20× column volume with buffer B (50 mM Tris-HCl, 300 mM NaCl, 20 mM imidazole, pH 8), and protein(s) eluted with buffer C (50 mM Tris-HCl, 300 mM NaCl, 200 mM imidazole, pH 8). MalE-tagged proteins were purified using amylose affinity purification following the recommendations of the manufacturer (New England Biolabs).

### Primer extension

The primer f2-rev, which is complementary to +88 to +107 of *fibA*, was labelled at the 5′-end using [γ-^32^P]-ATP (9.25 MBq, Amersham) using T4 polynucleotide kinase (New England Biolabs). 0.7 pmol of ^32^P-labelled f2-rev and 5 µg of total RNA isolated as described from exponentially growing DK1622 were mixed in 10 µl of hybridization buffer (50 mM HEPES, pH 7.0, 100 mM KCl) and incubated for 1 min at 70°C and slowly cooled to 45°C. Reverse transcription was initiated by adding 40 U M-MuLV reverse transcriptase (Fermentas) in 6 µl of extension buffer (110 mM Tris-HCl, pH 7.8, 10 mM MgCl_2_, 10 mM DTT 110 µM of each dNTP) followed by incubation at 42°C for 30 min. Reaction products were separated on a 6% DNA sequencing gel (Sambrook and Russell, 2001) and visualized by autoradiography.

### Electrophoretic mobility shift assays

The HEX-f2-labelled DNA fragment was generated by PCR using the primer f2-forw HEX-labelled at the 5′-end and unlabelled f2-rev. Reactions were carried out in buffer A [10 mM Tris-HCl pH 7.5, 50 mM KCl, 1 mM EDTA, 1 mM DTT, 50 ng µl^−1^ poly(dI-dC) (Sigma Aldrich)] in the presence of 2.5 nM probe (HEX-f2) and 4 µM MalE-DigR. Competitor DNA was added in 150-fold molar excess. Reactions were incubated at 25°C for 20 min. Samples were loaded on a 5% polyacrylamide gel, and electrophoresed in 0.5× TBE (Sambrook and Russell, 2001) at 4°C. The gel was pre-run in 0.5× TBE for 1 h. Gels were scanned for HEX signals on a Typhoon phosphorimager (GE Healthcare).

### DNase I footprinting experiments

The ^32^P-labelled f2 DNA fragment was generated by PCR using the primer f2-forw ^32^P-labelled at the 5′-end and unlabelled f2-rev. Labelled DNA fragment and DigR were mixed and incubated as described for EMSA in 80 µl reaction volumes. Subsequently, 20 µl of DNase I (New England Biolabs) mixture [0.1 u µl^−1^, in 5× DNase I buffer (50 mM MgCl_2_, 25 mM CaCl_2_, 500 mM NaCl, 200 mM Tris-HCl, pH 7.9, 1 mM DTT)] was added for 20 s at 37°C. Reactions were stopped by adding 100 µl of stop solution (20 mM EDTA pH 8.0, 0.3 µg µl^−1^ herring testes DNA). DNase digestion patterns were analysed on 6% DNA sequencing gel (Sambrook and Russell, 2001) and visualized by autoradiography.
