# Supplementary material for: The orphan histidine protein kinase SgmT is a c-di-GMP receptor and regulates composition of the extracellular matrix together with the orphan DNA binding response regulator DigR in Myxococcus xanthus
Source: Mol Microbiol. 2012 Mar 6;84(1):147–65. doi: 10.1111/j.1365-2958.2012.08015.x (PMC3509222; doi:10.1111/j.1365-2958.2012.08015.x)
Supplement: Supplementary file 1 [file mmi0084-0147-SD1.pdf]

Supplementary Information for

The orphan histidine protein kinase SgmT is a c-di-GMP receptor and regulates composition of the extracellular matrix together with the orphan DNA binding response regulator DigR in *Myxococcus xanthus*

Tobias Petters, Xin Zhang, Jutta Nesper[1], Anke Treuner-Lange, Nuria Gomez-Santos, Michael Hoppert[2], Urs Jenal[1] and Lotte Søgaard-Andersen[3]
Max Planck Institute for Terrestrial Microbiology,
Karl-von-Frisch Str. 10,
35043 Marburg, Germany

[1] Biozentrum of the University of Basel,
Klingelbergstrasse 50,
CH-4054 Basel, Switzerland

[2] Institute of Microbiology & Genetics,
Georg-August-Universität Göttingen,
37077 Göttingen, Germany

[3] Corresponding author
Tel. 49-6421-178201
Fax 49-6421-178209
E-mail: sogaard@staff.uni-marburg.de

This file includes:

Supplementary Tables S1-S4.

Supplementary Figures S1-S4.

References.

Table S1. Genes differentially expressed in *ΔsgmT* and *ΔdigR* mutants[a].

ID No.[b]	MXAN	Gene	Annotation	SA3502 Δ*sgmT* Expression ratio ± standard deviation		SA1804 Δ*digR* Expression ratio ± standard deviation		Localization signal[c]	Functional category	Phylogenetic distribution[d]
				Micro-arrays	qRT-PCR	Micro-arrays	qRT-PCR			
1	MXAN3175	-	hypothetical protein	-68.3 ± 0.8	-8364.1 ± 1597.3	-50.2 ± 0.4	-5983.1 ± 663.1	SpI	not defined	*M. xanthus*
2	MXAN0587	-	trypsin domain-containing protein	-21.5 ± 0.7	-406.4 ± 66.5	-22.5 ± 0.7	-393.4 ± 50.9	SpII	protease/peptidase	Conserved
3	MXAN6106	*fibA*	matrix-associated zinc metalloprotease FibA	-8.3 ± 1.0	-10.8 ± 0.9	-15.2 ± 0.9	-20.3 ± 4.1	SpII	protease/peptidase	Conserved
4	MXAN5033	-	hypothetical protein	-10.9 ± 0.6	-333.1 ± 27.7	-9.5 ± 0.8	-237.8 ± 1.0	SpII	not defined	*M. xanthus*
5	MXAN2856	-	type II lantibiotic, LanA homolog	-9.5 ± 0.8		-9.5 ± 0.7		CYT	antimicrobial	Myxococcales
6	MXAN4293	*dkxM*	hypothetical protein	-8.2 ± 1.0		-8.1 ± 0.9		CYT	secondary metabolism	Myxococcales
7	MXAN0504	-	hypothetical protein	-7.1 ± 0.6		-7.9 ± 0.5		SpI	not defined	*M. xanthus*
8	MXAN2855	-	type II lantibiotic, LanA homolog	-7.4 ± 0.9	-46.6 ± 4.2	-7.8 ± 0.6	-54.7 ± 4.6	CYT	antimicrobial	Conserved
9	MXAN4290	*dkxO*	putative thioesterase	-8.6 ± 0.8		-7.2 ± 0.9		CYT	secondary metabolism	Conserved
10	MXAN1650	-	trypsin domain-containing protein	-6.2 ± 0.9		-6.8 ± 0.7		SpI	protease/peptidase	Conserved
11	MXAN4291	-	putative acyl carrier protein	-6.6 ± 0.7		-5.8 ± 0.9		CYT	secondary metabolism	Conserved
12	MXAN5125	*mrpC*	transcriptional regulator MrpC	-6.1 ± 0.8		-5.1 ± 0.8		CYT	signal transduction	Conserved
13	MXAN4746	-	TonB-dependent receptor	-6.1 ± 0.7		-4.2 ± 0.6		SpI/OM	metabolism	Conserved
14	MXAN3964	-	trypsin domain-containing protein	-3.5 ± 0.9		-3.7 ± 1.6		SpII	protease/peptidase	Conserved
15	MXAN5169	-	hypothetical protein	-3.7 ± 0.9		-3.6 ± 0.7		CYT	not defined	*M. xanthus*
16	MXAN1672	-	hypothetical protein	-3.1 ± 0.9		-3.0 ± 1.1		SpII	not defined	*M. xanthus*
17	MXAN5040	-	aldehyde dehydrogenase family protein	-2.2 ± 0.8		-2.7 ± 0.9		CYT	energy production	Conserved
18	MXAN4421	-	putative lipoprotein	-2.1 ± 0.8		-2.1 ± 1.2		SpII	not defined	*M. xanthus*
1	MXAN6255	-	hypothetical protein	42.4 ± 1.5	156.5 ± 13.2	42.8 ± 1.2	183.1 ± 5.8	TMH	not defined	Conserved
2	MXAN5453	-	hypothetical protein	20.6 ± 1.4	60.8 ± 8.3	20.0 ± 1.5	93.3 ± 26.0	CYT	not defined	Conserved
3	MXAN3534	-	hypothetical protein	26.5 ± 1.5		15.4 ± 1.1		CYT	not defined	*M. xanthus*
4	MXAN3533	-	PheA/TfdB family FAD-binding monooxygenase	20.4 ± 1.3		8.9 ± 1.4		CYT	metabolism	Conserved
5	MXAN6254	-	hypothetical protein	7.9 ± 1.2		8.8 ± 1.4		TMH	not defined	Conserved
6	MXAN3971	-	putative lipoprotein, IF-tail domain	7.9 ± 1.2	7.6 ± 0.2	7.0 ± 1.2	7.3 ± 0.3	SpI	not defined	Conserved
7	MXAN1093	-	DNA-binding response regulator	6.6 ± 1.4	4.9 ± 0.2	5.7 ± 1.2	4.9 ± 0.3	CYT	signal transduction	Conserved

8	MXAN1676	-	oxidase, FAD binding	7.9 ± 1.4		5.6 ± 1.1		CYT	energy production	Conserved
9	MXAN4100	*hemB*	delta-aminolevulinic acid dehydratase	7.2 ± 1.3		5.3 ± 1.1		CYT	metabolism	Conserved
10	MXAN7212	-	hypothetical protein	4.9 ± 1.4		5.3 ± 1.2		SpII	not defined	Myxococcales
11	MXAN3564		M36 family peptidase	7.0 ± 1.3		5.2 ± 1.2		SpI	protease/peptidase	Conserved
12	MXAN3535	-	hypothetical protein	6.7 ± 1.6		4.9 ± 1.2		CYT	not defined	*M. xanthus*
13	MXAN7409	-	hypothetical protein	4.8 ± 1.5		4.8 ± 1.2		TMH	not defined	Conserved
14	MXAN6352	-	trypsin domain-containing protein	6.0 ± 1.4		4.6 ± 1.2		SpI	protease/peptidase	Conserved
15	MXAN7352	-	hypothetical protein	8.4 ± 1.4		4.5 ± 1.1		CYT	not defined	Myxococcales
16	MXAN6225	-	putative lipoprotein	5.3 ± 1.4		4.4 ± 1.2		SpII	not defined	Conserved
17	MXAN7351	-	hypothetical protein	7.4 ± 1.4		4.3 ± 1.2		SpI	not defined	Myxococcales
18	MXAN4541	-	hypothetical protein	7.0 ± 1.2		3.7 ± 1.2		TMH	not defined	*M. xanthus*
19	MXAN5845	-	hypothetical protein with CpxP domain	6.3 ± 1.2		3.6 ± 1.2		SpI	not defined	Conserved
20	MXAN7408	-	hypothetical protein	4.4 ± 1.6		3.5 ± 1.3		CYT	not defined	*M. xanthus*
21	MXAN6000	-	iron compound ABC transporter, periplasmic iron binding protein	4.0 ± 1.4		3.5 ± 1.2		SpI	metabolism	Conserved
22	MXAN6613	-	SdpC toxin-family protein	5.5 ± 1.4		3.5 ± 1.2		SpI	antimicrobial	Conserved
23	MXAN7353	-	putative beta-ketoacyl-acyl carrier protein synthase	5.7 ± 1.3		3.4 ± 1.2		CYT	metabolism	Conserved
24	MXAN6884	-	hypothetical protein	3.1 ± 1.4		3.3 ± 1.4		CYT	not defined	Myxococcales
25	MXAN6223	-	sensor histidine kinase	3.0 ± 1.3		3.1 ± 1.4		TMH	signal transduction	Conserved
26	MXAN7199	-	putative lipoprotein	3.7 ± 1.3		3.0 ± 1.2		SpI	not defined	Myxococcales
27	MXAN5300	-	Hypothetical	2.7 ± 1.2		3.0 ± 1.3		CYT	not defined	*M. xanthus*
28	MXAN1674	-	NAD dependent epimerase/dehydratase family protein	4.5 ± 1.3		2.9 ± 1.0		CYT	metabolism	Conserved
29	MXAN6534	-	PTS system mannose/fructose/sorbose family IIB subunit	3.2 ± 1.3		2.9 ± 1.1		CYT	metabolism	Conserved
30	MXAN3540	*sdhB*	succinate dehydrogenase iron-sulfur subunit	3.5 ± 1.3		2.9 ± 1.2		CYT	energy production	Conserved
31	MXAN7401	*vegA*	vegetative protein	3.2 ± 1.4		2.8 ± 1.2		CYT	not defined	Myxococcales
32	MXAN6227	-	putative lipoprotein	4.0 ± 1.2		2.8 ± 1.2		SpI	not defined	Conserved
33	MXAN4033	-	hypothetical protein	3.6 ± 1.3		2.8 ± 1.1		CYT	not defined	Myxococcales
34	MXAN0730	-	O-methyltransferase family protein	2.4 ± 1.2		2.8 ± 1.2		CYT	secondary metabolism	Conserved
35	MXAN5348	-	M23 peptidase domain-containing protein	2.1 ± 1.2		2.6 ± 1.2		TMH	protease/peptidase	Conserved
36	MXAN2812	-	hypothetical protein	3.1 ± 1.2		2.6 ± 1.2		CYT	not defined	Myxococcales

37	MXAN0522	-	putative lipoprotein	2.6 ± 1.3		2.5 ± 1.2		SpII	not defined	Myxococcales
38	MXAN5127	*phhA*	phenylalanine 4-monooxygenase	2.6 ± 1.3		2.5 ± 1.3		CYT	metabolism	Conserved
39	MXAN0982	-	hypothetical protein	2.5 ± 1.1		2.5 ± 1.1		SpI	not defined	Conserved
40	MXAN6981	-	hypothetical protein	2.3 ± 1.2		2.5 ± 1.2		CYT	not defined	Conserved
41	MXAN3539	*sdhA*	succinate dehydrogenase flavoprotein subunit	3.0 ± 1.3		2.5 ± 1.2		CYT	energy production	Conserved
42	MXAN5921	-	acyl-CoA dehydrogenase	2.5 ± 1.2		2.4 ± 1.2		CYT	metabolism	Conserved
43	MXAN7513	-	hypothetical protein	3.1 ± 1.4		2.4 ± 1.1		SpI	not defined	*M. xanthus*
44	MXAN1083	-	NADH dehydrogenase I, K subunit	2.4 ± 1.3		2.4 ± 1.3		TMH	energy production	Conserved
45	MXAN6393	*fabZ*	beta-hydroxyacyl-(acyl-carrier-protein) dehydratase FabZ	2.0 ± 1.2		2.3 ± 1.1		CYT	metabolism	Conserved
46	MXAN6886	-	putative lipoprotein	2.8 ± 1.3		2.3 ± 1.1		SpII	not defined	Conserved
47	MXAN2892	-	WGR domain-containing protein	2.3 ± 1.0		2.3 ± 1.1		CYT	not defined	Conserved
48	MXAN0212	-	aminotransferase, class I and II family protein	2.3 ± 0.8		2.3 ± 1.3		CYT	metabolism	Conserved
49	MXAN5160	-	DoxD-like family protein	2.1 ± 1.1		2.2 ± 1.2		TMH	not defined	Conserved
50	MXAN2661	-	5'-nucleotidase family protein	3.0 ± 1.3		2.2 ± 1.2		SpI	metabolism	Conserved
51	MXAN6086	*coxB*	cytochrome c oxidase, subunit II	2.5 ± 1.2		2.2 ± 1.2		TMH	energy production	Conserved
52	MXAN4038	*hisC*	histidinol-phosphate aminotransferase	2.8 ± 1.3		2.2 ± 1.2		CYT	metabolism	Conserved
53	MXAN7358	*tkt*	transketolase	2.2 ± 1.2		2.2 ± 1.2		CYT	metabolism	Conserved
54	MXAN2660	-	putative lipoprotein	2.5 ± 1.2		2.2 ± 1.3		SpII	not defined	Myxococcales
55	MXAN1084	-	NADH dehydrogenase I, J subunit	2.1 ± 0.8		2.2 ± 1.3		TMH	energy production	Conserved
56	MXAN1775	-	hypothetical membrane-protein	2.5 ± 1.4		2.2 ± 1.1		TMH	not defined	Conserved
57	MXAN6085	-	SCO1/SenC family protein	2.5 ± 1.2		2.2 ± 1.4		SpI	energy production	Conserved
58	MXAN6337	-	putative isocitrate dehydrogenase, NAD-dependent	2.9 ± 1.1		2.2 ± 1.2		CYT	energy production	Conserved
59	MXAN4176	-	outer membrane efflux protein	2.3 ± 1.3		2.2 ± 1.1		OM	drug resistence	Conserved
60	MXAN5836	-	M3 family peptidase	2.1 ± 0.8		2.1 ± 1.3		SpI	protease/peptidase	Conserved
61	MXAN1161	-	hypothetical protein	2.3 ± 1.2		2.1 ± 1.1		SpI	not defined	Conserved
62	MXAN6976	-	hypothetical protein	2.1 ± 0.7		2.1 ± 1.4		CYT	not defined	Myxococcales
63	MXAN0452	-	putative myo-inositol-1-phosphate synthase	2.5 ± 1.1		2.1 ± 1.3		SpI	metabolism	Conserved
64	MXAN0390	-	ribonuclease activity regulator protein RraA	2.1 ± 0.9		2.1 ± 1.1		CYT	metabolism	Conserved
65	MXAN2600	-	hypothetical protein	2.3 ± 1.3		2.1 ± 1.2		SpI	not defined	Myxococcales

66	MXAN4039	*accA*	acetyl-CoA carboxylase, carboxyl transferase, alpha subunit	2.5 ± 1.1		2.1 ± 1.2		CYT	metabolism	Conserved
67	MXAN5311	-	hypothetical protein	2.5 ± 1.3		2.1 ± 1.3		CYT	not defined	Myxococcales
68	MXAN6535	-	PTS system mannose/fructose/sorbose family IIA subunit	2.3 ± 1.3		2.1 ± 1.2		CYT	metabolism	Conserved
69	MXAN1954	*trxB*	thioredoxin-disulfide reductase	2.6 ± 1.2		2.1 ± 1.1		CYT	metabolism	Conserved
70	MXAN4619	-	glycosyl transferase, group 2 family protein	2.0 ± 1.2		2.1 ± 1.1		CYT	energy production	Conserved
71	MXAN3769	-	NUDIX family hydrolase	2.1 ± 1.1		2.1 ± 1.0		CYT	replication	Conserved
72	MXAN3519	*gltA*	citrate synthase	2.5 ± 1.3		2.1 ± 1.1		CYT	energy production	Conserved
73	MXAN6078	-	putative cytochrome c	2.3 ± 1.2		2.1 ± 1.4		SpI	energy production	Conserved
74	MXAN1807	-	excisionase family DNA-binding protein	2.8 ± 1.0		2.1 ± 1.1		CYT	transcription	Conserved
75	MXAN6783	-	decarboxylase, group II	2.0 ± 1.1		2.1 ± 1.1		CYT	metabolism	Conserved
76	MXAN0481	-	hypothetical protein	2.4 ± 1.0		2.1 ± 0.8		SpI	not defined	Conserved
77	MXAN2536	-	putative long-chain-fatty-acid--CoA ligase	2.4 ± 0.7		2.0 ± 1.1		CYT	metabolism	Conserved
78	MXAN0825	-	hypothetical protein	2.2 ± 1.2		2.0 ± 1.0		SpI	not defined	Conserved
79	MXAN0402	*atpB*	ATP synthase F0, A subunit	2.0 ± 0.8		2.0 ± 1.3		CYT	energy production	Conserved
80	MXAN5559	-	putative nitrate reductase, gamma subunit	2.1 ± 0.8		2.0 ± 1.2		TMH	energy production	Conserved
81	MXAN5577	*ppa*	inorganic pyrophosphatase	2.1 ± 1.2		2.0 ± 1.1		CYT	energy production	Conserved
82	MXAN0404	*atpF*	ATP synthase F0, B subunit	2.1 ± 0.7		2.0 ± 1.2		CYT	energy production	Conserved
83	MXAN6088	*coxC*	cytochrome c oxidase, subunit III	2.4 ± 1.3		2.0 ± 1.3		TMH	energy production	Conserved
84	MXAN2849	-	hypothetical protein	2.4 ± 1.2		2.0 ± 1.1		CYT	not defined	Conserved
85	MXAN6228	-	hypothetical protein	2.5 ± 1.2		2.0 ± 1.1		CYT	not defined	*M. xanthus*
86	MXAN6978	-	putative lipoprotein	2.9 ± 1.4		2.0 ± 1.3		SpII	not defined	Myxococcales
	MXAN0962	-	putative lipoprotein, von Willebrand factor type A domain	Not significant		-2.1 ± 0.9		SpII	not defined	Myxococcales
	MXAN5261	-	putative lipoprotein	Not significant		-2.1 ± 0.8		SpII	not defined	Myxococcales
	MXAN0277	-	hypothetical protein	Not significant		2.5 ± 2.1		CYT	not defined	Conserved
	MXAN0433	-	S-adenosylmethionine-dependent methyltransferase	Not significant		2.5 ± 1.2		CYT	secondary metabolism	Conserved
	MXAN2558	-	lipoprotein	Not significant		2.4 ± 1.4		SpII	outer membran protein	Conserved
	MXAN0703	-	hypothetical protein	Not significant		2.2 ± 1.0		SpI	not defined	Myxococcales
	MXAN2260	-	potassium efflux system family protein	Not significant		2.2 ± 1.1		TMH	metabolism	Conserved
	MXAN2967	-	RND efflux transporter	Not significant		2.2 ± 1.3		SpI	drug resistence	Conserved

	MXAN6686	-	putative lipoprotein	Not significant		2.2 ± 1.3		SpII	not defined	Myxococcales
	MXAN0824	-	Alpha/beta hydrolase fold family	Not significant		2.1 ± 1.2		CYT	metabolism	Conserved
	MXAN2338	*cobA*	uroporphyrin-III C-methyltransferase	Not significant		2.1 ± 1.2		CYT	metabolism	Conserved
	MXAN5806	-	putative glutamate-cysteine ligase	Not significant		2.1 ± 1.1		CYT	metabolism	Conserved
	MXAN6569	-	ferrichrome ABC transporter, ATP-binding protein	Not significant		2.1 ± 1.2		CYT	metabolism	Conserved
	MXAN6903	-	amidinotransferase family protein	Not significant		2.1 ± 1.3		CYT	metabolism	Conserved
	MXAN1781	-	glyoxalase family protein	Not significant		2.0 ± 1.1		CYT	metabolism	Conserved
	MXAN5830	-	hypothetical protein	Not significant		2.0 ± 1.1		CYT	not defined	Myxococcales
	MXAN7022	-	putative multidrug resistance protein NorM	Not significant		2.0 ± 1.1		TMH	drug resistence	Conserved
	MXAN0732	*rokA*	response regulator	2.7 ± 1.3		Not significant		CYT	signal transduction	Conserved
	MXAN4327	-	Glu/Leu/Phe/Val dehydrogenase family protein	2.7 ± 1.3		Not significant		CYT	metabolism	Conserved
	MXAN0805	-	peptidase, M10A/M12A subfamilies	2.6 ± 1.2		Not significant		SpII	protease/peptidase	Myxococcales
	MXAN1001	-	hypothetical protein	2.5 ± 1.1		Not significant		SpI	not defined	Myxococcales
	MXAN3850	-	putative general stress protein GsiB	2.5 ± 1.0		Not significant		CYT	not defined	Conserved
	MXAN5341	*frr*	ribosome recycling factor	2.5 ± 1.2		Not significant		CYT	translation	Conserved
	MXAN0873	-	adenylate/guanylate cyclase domain-containing protein	2.4 ± 1.0		Not significant		CYT	signal transduction	Conserved
	MXAN1264	*pckG*	phosphoenolpyruvate carboxykinase	2.4 ± 1.3		Not significant		CYT	energy production	Conserved
	MXAN1561	-	putative lipoprotein	2.4 ± 1.3		Not significant		CYT	not defined	*M. xanthus*
	MXAN3436		putative transcriptional regulator	2.4 ± 0.7		Not significant		CYT	transcription	Conserved
	MXAN5406	-	hypothetical protein	2.4 ± 1.2		Not significant		CYT	not defined	Conserved
	MXAN6782	-	selenocysteine-containing	2.4 ± 1.2		Not significant		CYT	not defined	*M. xanthus*
	MXAN0213		AsnC family transcriptional regulator	2.3 ± 1.3		Not significant		CYT	transcription	Conserved
	MXAN2232	-	pirin family protein	2.3 ± 1.3		Not significant		CYT	transcription	Conserved
	MXAN3191	-	diacylglycerol kinase catalytic subunit	2.3 ± 1.2		Not significant		CYT	metabolism	Myxococcales
	MXAN5340	-	response regulator/GGDEF domain-containing protein	2.3 ± 1.2		Not significant		CYT	signal transduction	Myxococcales
	MXAN5750	*carF*	carotenoid synthesis regulator CarF	2.3 ± 1.3		Not significant		TMH	secondary metabolism	Conserved
	MXAN6036	*sucB*	2-oxoglutarate dehydrogenase, E2 component, dihydrolipoamide succinyltransferase	2.3 ± 1.3		Not significant		CYT	energy production	Conserved
	MXAN0652	-	hypothetical protein	2.2 ± 1.0		Not significant		CYT	not defined	Conserved

	MXAN1071	*ssb*	single-strand binding protein	2.2 ± 1.2		Not significant	CYT	replication	Conserved
	MXAN2589	-	HesB/YadR/YfhF family protein, Fe-S cluster biogenesis	2.2 ± 1.2		Not significant	CYT	metabolism	Conserved
	MXAN4159	*udk*	uridine kinase	2.2 ± 1.2		Not significant	CYT	metabolism	Conserved
	MXAN4213	*ribA*	GTP cyclohydrolase II	2.2 ± 1.0		Not significant	CYT	metabolism	Conserved
	MXAN4769	*acpP*	acyl carrier protein	2.2 ± 1.2		Not significant	CYT	metabolism	Conserved
	MXAN6035	*sucA*	2-oxoglutarate dehydrogenase E1 component	2.2 ± 1.2		Not significant	CYT	energy production	Conserved
	MXAN6079	-	putative molybdopterin oxidoreductase, iron-sulfur binding subunit	2.2 ± 1.2		Not significant	CYT	energy production	Conserved
	MXAN6082	-	cytochrome c family protein	2.2 ± 1.2		Not significant	SpII	energy production	Conserved
	MXAN6224	-	Fis family DNA-binding response regulator	2.2 ± 1.1		Not significant	CYT	signal transduction	Conserved
	MXAN6513	-	NUDIX family hydrolase	2.2 ± 1.2		Not significant	CYT	replication	Conserved
	MXAN0236	*dnaN*	DNA polymerase III, beta subunit	2.1 ± 1.1		Not significant	CYT	replication	Conserved
	MXAN0763	-	response regulator	2.1 ± 1.1		Not significant	CYT	signal transduction	*M. xanthus*
	MXAN1982	-	putative lipoprotein	2.1 ± 1.1		Not significant	SpI	not defined	Myxococcales
	MXAN2024	-	glutaredoxin-like protein	2.1 ± 1.2		Not significant	CYT	defense mechanism	Conserved
	MXAN2479	*rho*	transcription termination factor Rho	2.1 ± 1.2		Not significant	CYT	transcription	Conserved
	MXAN2652	*trx*	thioredoxin	2.1 ± 1.2		Not significant	Cyt	defense mechanism	Conserved
	MXAN2729	-	NADH dehydrogenase I, D subunit	2.1 ± 1.2		Not significant	CYT	energy production	Conserved
	MXAN2960	*tatA*	twin arginine-targeting protein translocase	2.1 ± 1.2		Not significant	TMH	protein secretion	Conserved
	MXAN2968	-	RND family efflux transporter MFP subunit	2.1 ± 1.1		Not significant	SpII	drug resistence	Conserved
	MXAN3387	-	hypothetical protein	2.1 ± 1.1		Not significant	CYT	not defined	Conserved
	MXAN3764	-	peptidylprolyl cis-trans isomerase, cyclophilin-type	2.1 ± 1.2		Not significant	CYT	protein modification	Conserved
	MXAN4054	*clpX*	ATP-dependent protease ATP-binding subunit ClpX	2.1 ± 1.2		Not significant	CYT	protein modification	Myxococcales
	MXAN5752	-	hypothetical protein	2.1 ± 1.2		Not significant	SpII	not defined	Myxococcales
	MXAN6190	-	solute/sodium symporter (SSS) family protein	2.1 ± 1.2		Not significant	TMH	metabolism	Conserved
	MXAN6830	*panD*	aspartate 1-decarboxylase	2.1 ± 1.3		Not significant	CYT	metabolism	Conserved
	MXAN0855	-	putative chemotaxis protein MotB	2.0 ± 1.1		Not significant	SpII	outer membran protein	Conserved
	MXAN2666	*pdhA*	pyruvate dehydrogenase complex, E1 component	2.0 ± 1.2		Not significant	CYT	energy production	Conserved
	MXAN3012	*hslV*	ATP-dependent protease peptidase subunit	2.0 ± 1.1		Not significant	CYT	protein modification	Conserved

	MXAN5703	-	PspA/IM30 family protein	2.0 ± 1.2		Not significant		CYT	transcription	Conserved
	MXAN2791[e]	*prtB*	protease B	Not significant	-1.7 ± 0.0	Not significant	-2.1 ± 0.1	SpII	protease/peptidase	Conserved
	MXAN5786[e]	*pilC*	type 4 fimbrial assembly protein PilC	Not significant	-1.4 ± 0.0	Not significant	-1.2 ± 0.1	TMH	cell motility	Conserved
	MXAN6694[e]	*difC*	fibril biogenesis regulator DifC	Not significant	-1.5 ± 0.1	Not significant	-1.1 ± 0.2	CYT	signal transduction	Conserved
	MXAN7415[e]	*epsZ*	sugar transferase	Not significant	1.8 ± 0.1	Not significant	2.0 ± 0.3	SpI	cell envelope biogenesis	Conserved

[a] Expression ratios are calculated as the expression in the Δ*digR* or Δ*sgmT* mutants over the expression in wild type. A negative expression ratio corresponds to a gene that is more highly expressed in the wild type than in the Δ*sgmT* or Δ*digR* mutants. A positive expression ratio corresponds to a gene that is more highly expressed in Δ*digR* or Δ*sgmT* mutants than in wild type. Errors represent the standard deviation. Genes highlighted in red (decreased expression in Δ*sgmT* and Δ*digR* mutants) and blue (increased expression in Δ*sgmT* and Δ*digR* mutants) contain a DigR binding site in their promoter region (or if part of a potential operon, in the promoter region of the operon).

[b] Identification numbers indicate the 18 genes with decreased and 86 genes with increased expression that are significantly regulated in Δ*digR* and Δ*sgmT* mutants and were analyzed in Fig.3. According to their identification number genes were plotted in Fig.3B. Genes without ID number are significantly regulated only in Δ*digR* or Δ*sgmT* mutants.

[c] Localization signals are indicated as follows: SpI, signal peptide type I; SpII signal peptide type II; TMH, trans-membrane helix; OM, outer membrane; CYT, no localization signal, i.e. no signal peptide, no twin-arginine signal sequence, no trans-membrane helix and no homology to outer membrane proteins. Localization signals were identified using SignalP3 (Emanuelsson *et al.*, 2007) and TMHMM2 (Krogh *et al.*, 2001).

[d] Phylogenetic distribution of proteins is as follows: Proteins labelled conserved have orthologs in least one species outside the Myxococcales; proteins labelled Myxococcales have orthologs in at least two of the four Myxococcales species with completely sequenced genomes (*Anaeromyxobacter dehalogenans, Stigmatella aurantiaca*, *Sorangium cellulosum,* and *M. xanthus*; proteins labelled *M. xanthus* have no orthologs outside *M. xanthus*; data from (Huntley *et al.*, 2011).

[e] These four genes served as controls in the qRT-PCR analysis.

Table S2. Promoters similarly regulated by SgmT and DigR and containing a DigR binding site

SgmT/DigR regulated gene	In operon with[a]	Directly upstream of DigR regulated gene			Directly upstream of first gene in operon		
		-35[b]	-10[b]	DigR binding site[c]	-35[b]	-10[b]	DigR binding site[c]
**Activated by SgmT/DigR**							
MXAN0504	-	Yes	No	Upstream -35	-	-	-
MXAN1672	-	No	Yes	Between -35 and -10	-	-	-
MXAN2855	2857-2852	NA	NA	NA	Yes	No	Upstream -35 of MXAN2857
MXAN2856	2857-2852	NA	NA	NA	Yes	No	Upstream -35 of MXAN2857
MXAN3175	-	Yes	Yes	Upstream -35	-	-	-
MXAN4421	-	Yes	Yes	Upstream -35	-	-	-
MXAN4746	-	Yes	No	Between -35 and -10	-	-	-
MXAN6106	-	Yes	No	One upstream of -35<br>One downstream of -10	-	-	-
**Repressed by SgmT/DigR**							
MXAN1676	-	Yes	Yes	Downstream of ATG	-	-	-
MXAN5453	-	Yes	No	Between -35 and -10	-	-	-
MXAN6254	6254-6255	Yes	Yes	Overlap with -10	-	-	-
MXAN6255	6254-6255	NA	NA	NA	Yes	Yes	Overlap with -10 of MXAN6254
MXAN7212	-	Yes	No	Between -35 and -10	-	-	-
MXAN7351	7353-7350	NA	NA	NA	Yes	Yes	Overlap with -10 of MXAN7353
MXAN7352	7353-7350	NA	NA	NA	Yes	Yes	Overlap with -10 of MXAN7353
MXAN7353	7353-7350	Yes	Yes	Overlap with -10	-	-	-

[a] If the distance between ORFs transcribed in the same direction was less than 100 bp, they were considered to be in an operon. First gene in an operon is underlined.
[b] Yes: Three or more nucleotides conserved with *E. coli* consensus promoter sequences: -35 TTGACA or -10 TATAAT; No: Less than three nucleotides conserved with *E. coli* consensus promoter sequences: -35 TTGACA or -10 TATAAT; NA: Not applicable
[c] DigR binding sites were searched ±200 bp from a translational start codon using the palindromic part of the DigR binding site in the *fibA* promoter (**TAATC**-N14-**GATTA**) and allowing four mismatches. All promoters with decreased expression in the absence of SgmT and DigR were screened for DigR binding sites. Only promoters with more than a 4.0-fold increase in the absence of DigR were screened for a DigR binding site. Note that MXAN7353 is less than 4.0-fold reduced in expression in the absence of DigR. It is included here because it is likely in an operon with MXAN7351 and MXAN7352.

Table S3. Sequences of promoters containing DigR binding sites

SgmT/DigR activated promoters	Promoter element[a]				
	-35	Spacer	-10	Distance from -10 to translation start*	DigR binding site[a, b]
MXAN0504	TTGACA	17	CACCTT	-57	-137 - TAAGCACAATCACGTACGCGTTTC
MXAN1672	TTCCGT	15	TACCGT	-39	-59 - TATCGGAACACTTATTACCGTTTA
MXAN2857	TTGACA	17	TCATCG	-73	-158 - AAATCACTCCCACCGTCCCGCTTG
MXAN3175	TTGAAC	18	TAGTAA	-47	-120 - CAATCATCCATATCCACTGAATTA
MXAN4421	TTGCGC	17	TCCCAT	-44	-166 - TCATCTTCGGCGCCCTGTCGCTGT
MXAN4746	TTGACG	17	TGACGA	-61	-81 - CAATCGTTGAGCCAGTGACGATTC
MXAN6106	TTGAAA	17	TTACCT	-33	-119 - TAATCGTAAATAACCTGAAATCTA
					-25 - TAATCTTTCCGCTTAGGAGGATTA
**SgmT/DigR repressed promoters**					
MXAN1676	TTCATC	16	CATGAC	-140	+139 - GAATCACTCGAAGGCTGGGGCTTC
MXAN5453	TTGTAA	19	TCGTGT	-23	-50 - TAATCAACGACGCTCCTGCTAATC
MXAN6254	TTCACC	17	TCTCAT	-28	-31 - TCATCGAAAAGACACATGCGATTG
MXAN7212	TTCAAA	18	TGCGAG	-105	-131 - AAATCCCGGCCCCGAGAAGAATGC
MXAN7353	TTCGTA	16	CTCAAT	-60	-40 - CAATCCCCCGTCGCCAGGGAATCC

[a] All coordinates are with respect to the suggested translational start codon.
[b] See Table S2 footnote [c]. Matches to the search sequence are underlined.

Table S4. *M. xanthus* proteins predicted to be involved in c-di-GMP metabolism and regulation[a]

Gene	I-site	A-site	EAL motif	HD-GYP motif	Domain structure[b]	Ref.
**GGDEF domain proteins**						
MXAN1525	RREQ	GGEEF	NA	NA	FHA-GGDEF	
MXAN2643	QNLD	GGDEF	NA	NA	GAF-GGDEF	
MXAN2997	RKVD	GGEEF	NA	NA	sp-RPT-HAMP-GAF-GGDEF	
MXAN3213/actA	RDAD	GDCQF	NA	NA	REC-GGDEF	(Gronewold & Kaiser, 2001)
MXAN3705	RDTD	GGEEF	NA	NA	sp-4TMH-GAF-GAF-GGDEF	
MXAN3735	RESD	GGDEF	NA	NA	REC-GGDEF	
MXAN4029	RRED	GGEEF	NA	NA	FHA-GGDEF	
MXAN4257	VPGG	EGGAF	NA	NA	REC-GAF-PAS-GGDEF	
MXAN4445	APGD	AGDDF	NA	NA	REC-GAF-PAS-HK-REC-REC-GGDEF	
MXAN4463	RDHD	GGDEY	NA	NA	REC-GGDEF	
MXAN4640/sgmT	RHPD	GGGVF	NA	NA	GAF-HK-REC-GGDEF	
MXAN5053	RDSD	SDQEF	NA	NA	REC-GAF-GGDEF	
MXAN5199	RTED	GGEEF	NA	NA	FHA-GGDEF	
MXAN5340	RDID	ADSRF	NA	NA	REC-GGDEF	
MXAN5366	RETD	GGEEF	NA	NA	REC-GGDEF	
MXAN5791	REVD	GGEEF	NA	NA	REC-GGDEF	
MXAN7362	RRED	GGEEF	NA	NA	REC-HPt-REC-REC-GGDEF	
**EAL domain proteins**						
MXAN2424	NA	NA	EAL	NA	EAL	
MXAN2530	NA	NA	EVL	NA	EAL	
**HD-GYP domain proteins**						
MXAN2061	NA	NA	NA	HD GYP	sp-HAMP-HD_GYP	
MXAN2807	NA	NA	NA	HD GTP	GSPII_E_N-HD_GYP-REC	
MXAN4232	NA	NA	NA	HD GYP	REC-HD_GYP	
MXAN4675	NA	NA	NA	HD GYP	REC-HD_GYP	
MXAN6298	NA	NA	NA	HD GYP	GAF-HD_GYP	

[a] NA, not applicable; proteins included are predicted to contain the indicated domains based on searches for conserved domains using SMART (Letunic *et al.*, 2004) and BLAST (Altschul *et al.*, 1997). I-sites, A-sites and EAL and HD-GYP motifs were identified based on sequence alignments.

[b] Abbreviations: FHA/fork head associated domain; REC/receiver; TMH/trans-membrane helix; HK/histidine kinase; Hpt/phosphotransfer domain; SP/signal peptide.

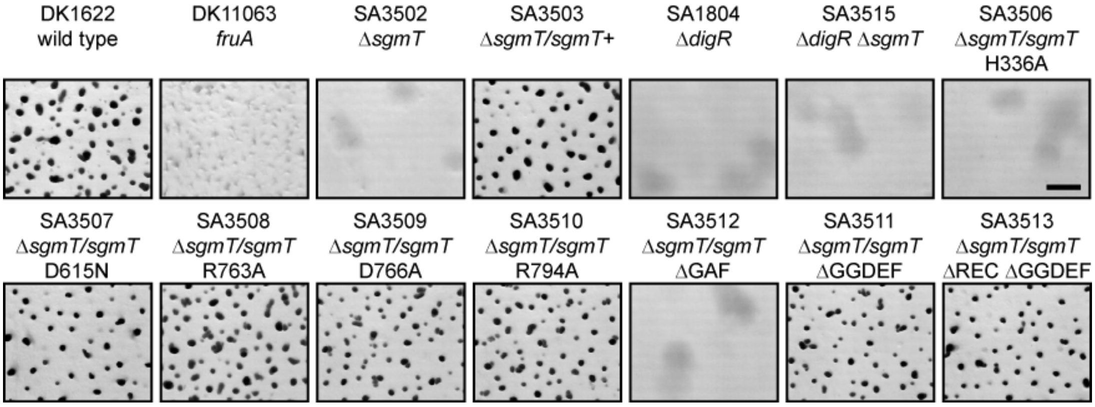

Figure S1. Starvation induced fruiting body formation by Δ*sgmT* and Δ*digR* mutants. Strains were incubated at 32 °C for 120h on TPM agar and visualized with a Leica MZ86 stereomicroscope. DK11063 carries a *fruA* mutation and served as a negative control. Scale bar, 500 µm.

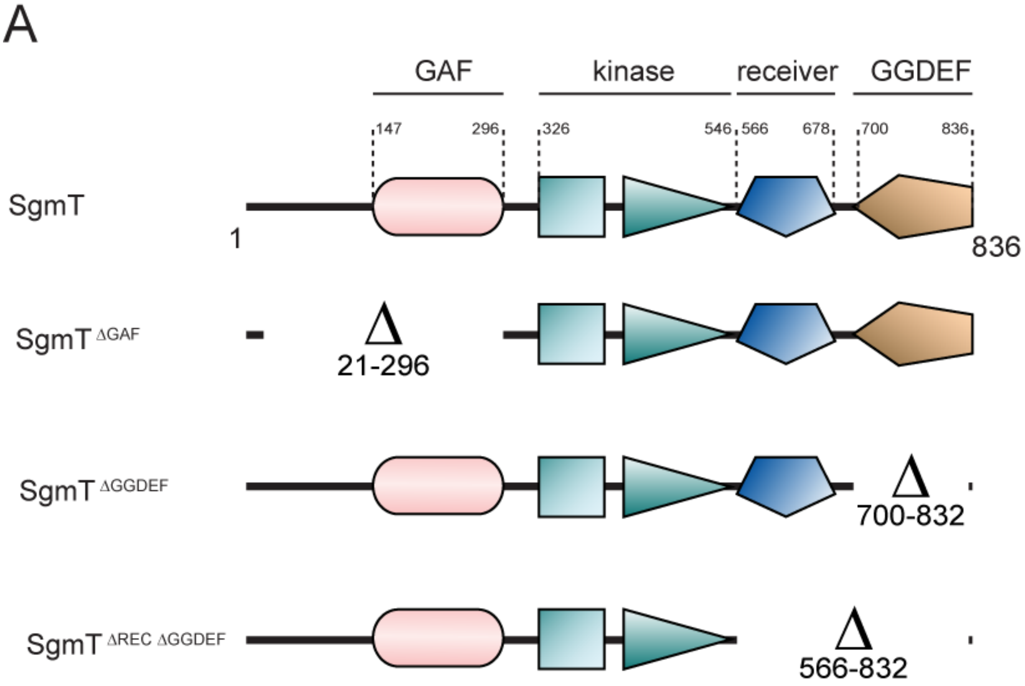

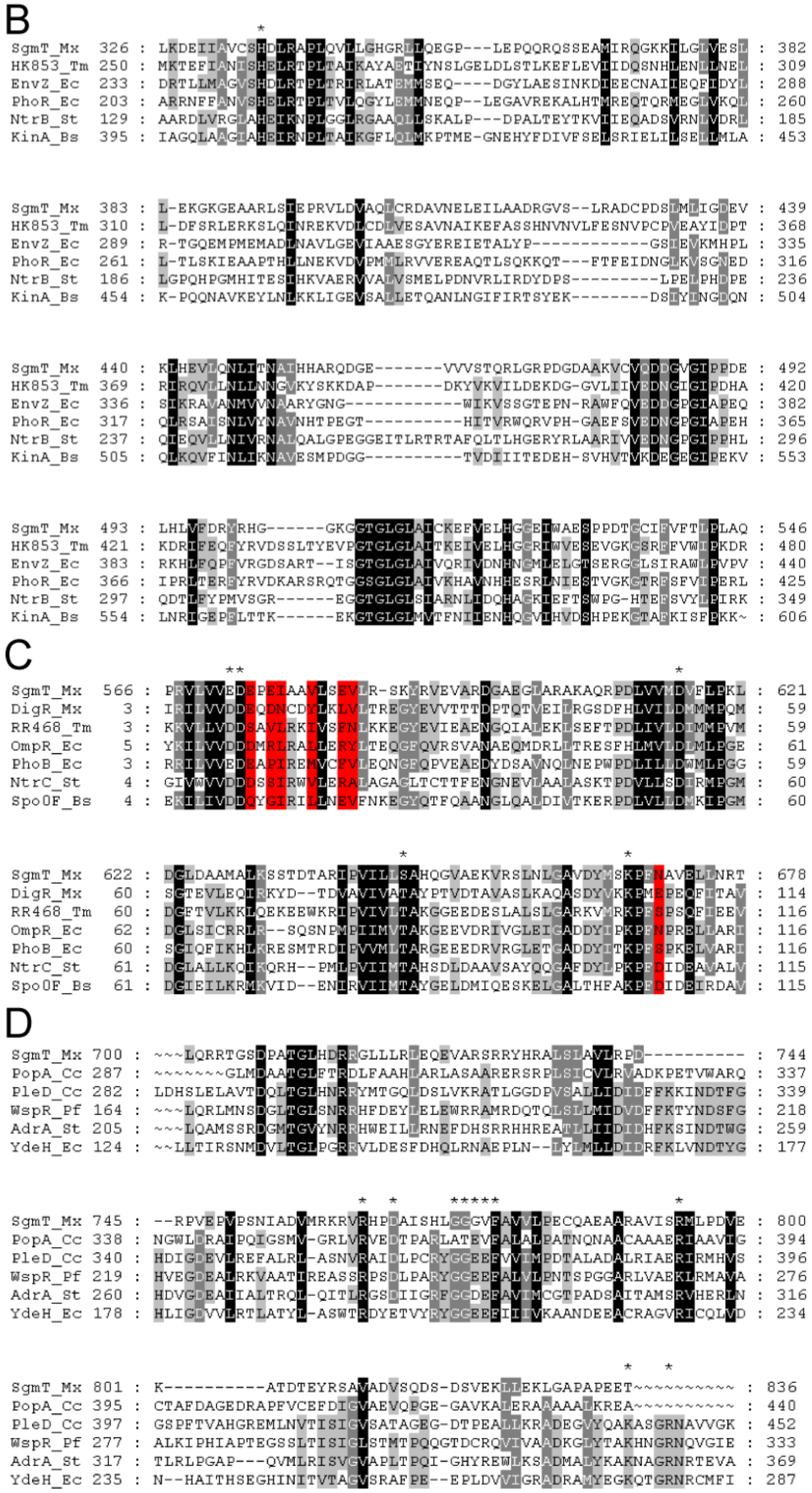
B
SgmT_Mx 326 : LKDEIIAVCSHDLRAPLQVLLGHGRLLQEGP---LEPQQRQSSEAMIRQGKKILGLVESL : 382
HK853_Tm 250 : MKTEFIANISHELRTPLTAIKAYAETIYNSLGELDLSTLKEFLEVIIDQSNHLENLLNEL : 309
EnvZ_Ec 233 : DRTLLMAGVSHDLRTPLTRIRLATEMMSEQ----DGYLAESINKDIEECNAIIEQFIDYL : 288
PhoR_Ec 203 : ARRNFFANVSHELRTPLTVLQGYLEMMNEQP--LEGAVREKALHTMREQTQRMEGLVKQL : 260
NtrB_St 129 : AARDLVRGLAHEIKNPLGGLRGAAQLLSKALP---DPALTEYTKVIIEQADSVRNLVDRL : 185
KinA_Bs 395 : IAGQLAAGIAHEIRNPLTAIKGFLQLMKPTME-GNEHYFDIVFSELSRIELILSELLMLA : 453
SgmT_Mx 383 : L-EKGKGEAARLSIEPRVLDVAQLCRDAVNELEILAADRGVS--LRADCPDSLMLIGDEV : 439
HK853_Tm 310 : L-DFSRLERKSLQINREKVDLCDLVESAVNAIKEFASSHNVNVLFESNVPCPVEAYIDPT : 368
EnvZ_Ec 289 : R-TGQEMPMEMADLNAVLGEVIAAESGYEREIETALYP------------GSIEVKMHPL : 335
PhoR_Ec 261 : L-TLSKIEAAPTHLLNEKVDVPMMLRVVEREAQTLSQKKQT---FTFEIDNGLKVSGNED : 316
NtrB_St 186 : LGPQHPGMHITESIHKVAERVVALVSMELPDNVRLIRDYDPS---------LPELPHDPE : 236
KinA_Bs 454 : K-PQQNAVKEYLNLKKLIGEVSALLETQANLNGIFIRTSYEK--------DSIYINGDQN : 504
SgmT_Mx 440 : KLHEVLQNLITNAIHHARQDGE-------VVVSTQRLGRPDGDAAKVCVQDDGVGIPPDE : 492
HK853_Tm 369 : RIRQVLLNLLNNGVKYSKKDAP-------DKYVKVILDEKDG-GVLIIVEDNGIGIPDHA : 420
EnvZ_Ec 336 : SIKRAVANMVVNAARYGNG------------WIKVSSGTEPN-RAWFQVEDDGPGIAPEQ : 382
PhoR_Ec 317 : QLRSAISNLVYNAVNHTPEGT----------HITVRWQRVPH-GAEFSVEDNGPGIAPEH : 365
NtrB_St 237 : QIEQVLLNIVRNALQALGPEGGEITLRTRTAFQLTLHGERYRLAARIVVEDNGPGIPPHL : 296
KinA_Bs 505 : QLKQVFINLIKNAVESMPDGG----------TVDIIITEDEH-SVHVTVKDEGEGIPEKV : 553
SgmT_Mx 493 : LHLVFDRYRHG------GKGGTGLGLAICKEFVELHGGEIWAESPPDTGCIFVFTLPLAQ : 546
HK853_Tm 421 : KDRIFEQFYRVDSSLTYEVPGTGLGLAITKEIVELHGGRIWVESEVGKGSRFFVWIPKDR : 480
EnvZ_Ec 383 : RKHLFQPFVRGDSART--ISGTGLGLAIVQRIVDNHNGMLELGTSERGGLSIRAWLPVPV : 440
PhoR_Ec 366 : IPRLTERFYRVDKARSRQTGGSGLGLAIVKHAVNHHESRLNIESTVGKGTRFSFVIPERL : 425
NtrB_St 297 : QDTLFYPMVSGR------EGGTGLGLSIARNLIDQHAGKIEFTSWPG-HTEFSVYLPIRK : 349
KinA_Bs 554 : LNRIGEPFLTTK------EKGTGLGLMVTFNIIENHQGVIHVDSHPEKGTAFKISFPKK~ : 606
C
SgmT_Mx 566 : PRVLVVEDEPEIAAVLSEVLR-SKYRVEVARDGAEGLARAKAQRPDLVVMDVFLPKL : 621
DigR_Mx 3 : IRILVVDDEQDNCDYLKLVLTREGYEVVTTTDPTQTVEILRGSDFHLVILDMMMPQM : 59
RR468_Tm 3 : KKVLLVDDSAVLRKIVSFNLKKEGYEVIEAENGQIALEKLSEFTPDLIVLDIMMPVM : 59
OmpR_Ec 5 : YKILVVDDDMRLRALLERYLTEQGFQVRSVANAEQMDRLLTRESFHLMVLDLMLPGE : 61
PhoB_Ec 3 : RRILVVEDEAPIREMVCFVLEQNGFQPVEAEDYDSAVNQLNEPWPDLILLDWMLPGG : 59
NtrC_St 4 : GIVWVVDDDSSIRWVLERALAGAGLTCTTFENGNEVLAALASKTPDVLLSDIRMPGM : 60
Spo0F_Bs 4 : EKILIVDDQYGIRILLNEVFNKEGYQTFQAANGLQALDIVTKERPDLVLLDMKIPGM : 60
SgmT_Mx 622 : DGLDAAMALKSSTDTARIPVILLSAHQGVAEKVRSLNLGAVDYMSKPFNAVELLNRT : 678
DigR_Mx 60 : SGTEVLEQIRKYD--TDVAVIVATAYPTVDTAVASLKAQASDYVKKPMEPEQFITAV : 114
RR468_Tm 60 : DGFTVLKKLQEKEEWKRIPVIVLTAKGGEEDESLALSLGARKVMRKPFSPSQFIEEV : 116
OmpR_Ec 62 : DGLSICRRLR--SQSNPMPIIMVTAKGEEVDRIVGLEIGADDYIPKPFNPRELLARI : 116
PhoB_Ec 60 : SGIQFIKHLKRESMTRDIPVVMLTARGEEEDRVRGLETGADDYITKPFSPKELVARI : 116
NtrC_St 61 : DGLALLKQIKQRH--PMLPVIIMTAHSDLDAAVSAYQQGAFDYLPKPFDIDEAVALV : 115
Spo0F_Bs 61 : DGIEILKRMKVID--ENIRVIIMTAYGELDMIQESKELGALTHFAKPFDIDEIRDAV : 115
D
SgmT_Mx 700 : ~~~LQRRTGSDPATGLHDRRGLLLRLEQEVARSRRYHRALSLAVLRPD---------- : 744
PopA_Cc 287 : ~~~~~~~GLMDAATGLFTRDLFAAHLARLASAARERSRPLSICVLRVADKPETVWARQ : 337
PleD_Cc 282 : LDHSLELAVTDQLTGLHNRRYMTGQLDSLVKRATLGGDPVSALLIDIDFFKKINDTFG : 339
WspR_Pf 164 : ~~~LQRLMNSDGLTGLSNRRHFDEYLELEWRRAMRDQTQLSLLMIDVDFFKTYNDSFG : 218
AdrA_St 205 : ~~~LQAMSSRDGMTGVYNRRHWEILLRNEFDHSRRHHREATLLIIDIDHFKSINDTWG : 259
YdeH_Ec 124 : ~~LLTIRSNMDVLTGLPGRRVLDESFDHQLRNAEPLN--LYLMLLDIDRFKLVNDTYG : 177
SgmT_Mx 745 : --RPVEPVPSNIADVMRKRVRHPDAISHLGGGVFAVVLPECQAEAARAVISRMLPDVE : 800
PopA_Cc 338 : NGWLDRAIPQIGSMV-GRLVRVEDTPARLATEVFALALPATNQNAACAAAERIAAVIG : 394
PleD_Cc 340 : HDIGDEVLREFALRL-ASNVRAIDLPCRYGGEEFVVIMPDTALADALRIAERIRMHVS : 396
WspR_Pf 219 : HVEGDEALRKVAATIREASSRPSDLPARYGGEEFALVLPNTSPGGARLVAEKLRMAVA : 276
AdrA_St 260 : HDVGDEAIIALTRQL-QITLRGSDIIGRFGGDEFAVIMCGTPADSAITAMSRVHERLN : 316
YdeH_Ec 178 : HLIGDVVLRTLATYL-ASWTRDYETVYRYGGEEFIIIVKAANDEEACRAGVRICQLVD : 234
SgmT_Mx 801 : K----------ATDTEYRSAVADVSQDS-DSVEKLLEKLGAPAPEET~~~~~~~~~~ : 836
PopA_Cc 395 : CTAFDAGEDRAPFVCEFDIGVAEVQPGE-GAVKALERAAAAALKREA~~~~~~~~~~ : 440
PleD_Cc 397 : GSPFTVAHGREMLNVTISIGVSATAGEG-DTPEALLKRADEGVYQAKASGRNAVVGK : 452
WspR_Pf 277 : ALKIPHIAPTEGSSLTISIGLSTMTPQQGTDCRQVIVAADKGLYTAKHNGRNQVGIE : 333
AdrA_St 317 : TLRLPGAP---QVMLRISVGVAPLTPQI-GHYREWLKSADMALYKAKNAGRNRTEVA : 369
YdeH_Ec 235 : N--HAITHSEGHINITVTAGVSRAFPE--EPLDVVIGRADRAMYEGKQTGRNRCMFI : 287

Figure S2. Domain organization of SgmT.

A. Schematic representation of the domain organization of SgmT. Constructs used in genetic experiments are shown.

B. Alignment of SgmT kinase domain with other kinase domains. Asterisk indicate the conserved phosphorylatable His336.

C. Alignment of SgmT receiver domain with receiver domain of DigR as well as other receiver domains. Asterisks indicate conserved signature residues of receiver domains (Stock *et al.*, 2000). Red indicates residues suggested to be involved in specific contacts to the kinase domain (Capra *et al.*, 2010).

D. Alignment of SgmT GGDEF domain with characterized GGDEF domains. Asterisks indicate I-site (consensus: RXXD), A-site (consensus: GGDEF), the secondary I-site (R) and two positively charged residues (K442 and R446 in PleD) that in active enzymes coordinate β- and γ-phosphate binding. Residues on black, dark grey, and light grey are conserved 100%, 80% and 60%, respectively. Alignments were generated using CLUSTAL (Thompson *et al.*, 1997).

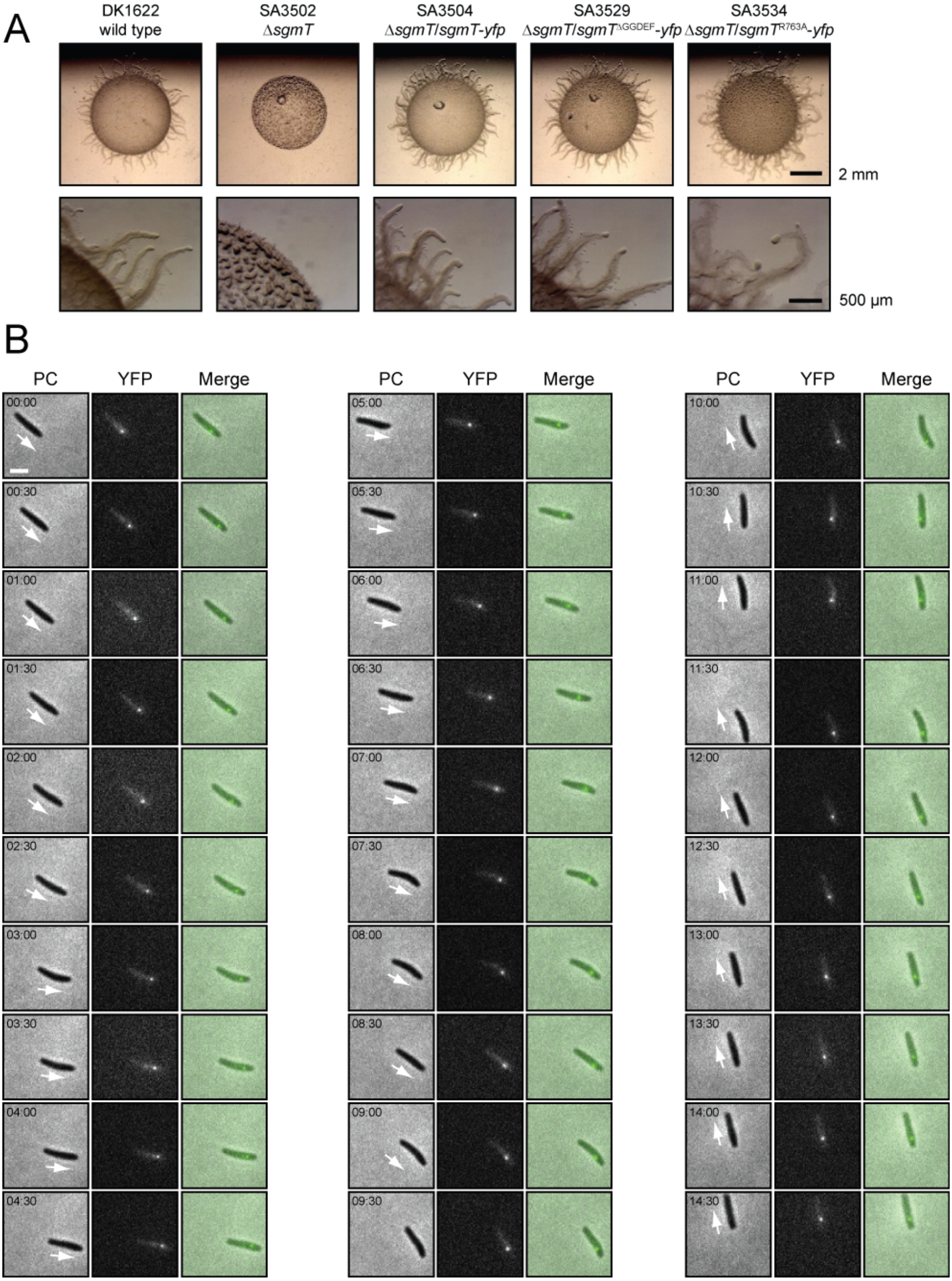
A
DK1622
wild type
SA3502
ΔsgmT
SA3504
ΔsgmT/sgmT-yfp
SA3529
ΔsgmT/sgmTΔGGDEF-yfp
SA3534
ΔsgmT/sgmTR763A-yfp
2 mm
500 µm
B
PC
YFP
Merge
00:00
00:30
01:00
01:30
02:00
02:30
03:00
03:30
04:00
04:30
05:00
05:30
06:00
06:30
07:00
07:30
08:00
08:30
09:00
09:30
10:00
10:30
11:00
11:30
12:00
12:30
13:00
13:30
14:00
14:30

Figure S3. Characterization of SgmT-YFP variants.

A. Motility phenotype of strains containing various derivatives of SgmT-YFP. Strains were incubated at 32 °C for 24 h on 0.5% agar supplemented with 0.5% CTT, and visualized with a Leica MZ86 stereomicroscope. Scale bars as indicated.

B. SgmT-YFP localizes to stationary cluster. Cells were transferred from exponentially growing cultures to a thin 1.0% agar pad on a microscope slide, and imaged by fluorescence- and phase-contrast (PC) microscopy every 30 sec for 15 min. Left, middle and right panels show phase-contrast, fluorescence and merged images, respectively. Arrows indicate the direction of movement. The cell reversed at 10:00 min. Scale bar, 2 μm.

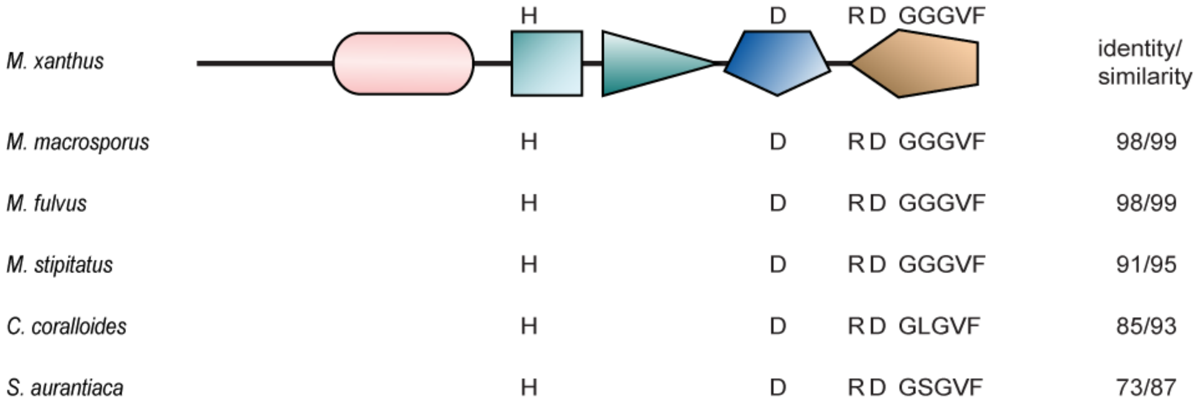

Figure S4. Domain structure and conserved sequences in SgmT homologs. The SgmT homologs in all six myxobacteria have the same domain structure and contain the conserved, phosphorylatable His residue (H) in the kinase domain, the Asp residue (D) in the receiver domain, the intact I-site (RxxD) and a degenerate A-site (GGGVF or variants). Numbers indicate % identity and % similarity between the *M. xanthus* protein and homologs.

References

Altschul, S. F., T. L. Madden, A. A. Schaffer, J. Zhang, Z. Zhang, W. Miller & D. J. Lipman, (1997) Gapped BLAST and PSI-BLAST: a new generation of protein database search programs. *Nucleic Acids Res.* **25**: 3389-3402.

Capra, E. J., B. S. Perchuk, E. A. Lubin, O. Ashenberg, J. M. Skerker & M. T. Laub, (2010) Systematic dissection and trajectory-scanning mutagenesis of the molecular interface that ensures specificity of two-component signaling pathways. *PLoS Genet.* **6**: e1001220.

Emanuelsson, O., S. Brunak, G. von Heijne & H. Nielsen, (2007) Locating proteins in the cell using TargetP, SignalP and related tools. *Nat. Protoc.* **2**: 953-971.

Gronewold, T. M. A. & D. Kaiser, (2001) The *act* operon controls the level and time of C-signal production for *Myxococcus xanthus* development. *Mol. Microbiol.* **40**: 744-756.

Huntley, S., N. Hamann, S. Wegener-Feldbrügge, A. Treuner-Lange, M. Kube, R. Reinhardt, S. Klages, R. Müller, C. M. Ronning, W. C. Nierman & L. Søgaard-Andersen, (2011) Comparative genomic analysis of fruiting body formation in Myxococcales. *Mol. Biol. Evol.* **28**: 1083-1097.

Krogh, A., B. Larsson, G. von Heijne & E. L. Sonnhammer, (2001) Predicting transmembrane protein topology with a hidden Markov model: application to complete genomes. *J Mol Biol* **305**: 567-580.

Letunic, I., R. R. Copley, S. Schmidt, F. D. Ciccarelli, T. Doerks, J. Schultz, C. P. Ponting & P. Bork, (2004) SMART 4.0: towards genomic data integration. **32 Database issue**: D142-D144.

Stock, A. M., V. L. Robinson & P. N. Goudreau, (2000) Two-component signal transduction. *Annu. Rev. Biochem.* **69**: 183-215.

Thompson, J. D., T. J. Gibson, F. Plewnik, F. Jeanmougin & D. G. Higgins, (1997) The ClustalX windows interface: flexible strategies for multiple sequence alignment aided by quality analysis tools. *Nucl. Acids. Res.* **24**: 4876-4882.
